# Supplementary material for: Delaying carbon dioxide removal in the European Union puts climate targets at risk
Source: Nat Commun. 2021 Nov 11;12:6490. doi: 10.1038/s41467-021-26680-3 (PMC8586243; doi:10.1038/s41467-021-26680-3)
Supplement: Supplementary file 1 — Editor Summary [file 41467_2021_26680_MOESM1_ESM.docx]

The implications of delaying carbon dioxide removal (CDR) are poorly understood. Here the authors highlight the potential extra costs and reduced removal potential of delayed CDR action, with a special focus on direct air capture and bioenergy with carbon capture and storage (DACCS and BECCS).
