# Supplementary material for: Delaying carbon dioxide removal in the European Union puts climate targets at risk
Source: Nat Commun. 2021 Nov 11;12:6490. doi: 10.1038/s41467-021-26680-3 (PMC8586243; doi:10.1038/s41467-021-26680-3)
Supplement: Supplementary file 2 — Supplementary Information File [file 41467_2021_26680_MOESM2_ESM.pdf]

Supplementary information

Delaying carbon dioxide removal in the European Union puts climate targets at risk

Ángel Galán-Martín[a,b,c,†], Daniel Vázquez[a,d,†], Selene Cobo[a], Niall Mac Dowell[e,f], José Antonio Caballero[d], Gonzalo Guillén-Gosálbez[a,*]

[a]*Institute for Chemical and Bioengineering, Department of Chemistry and Applied Biosciences, ETH Zürich, Vladimir-Prelog-Weg 1, 8093 Zürich, Switzerland*

[b]*Department of Chemical, Environmental and Materials Engineering, University of Jaén, Campus Las Lagunillas s/n, 23071 Jaén, Spain*

[c]*Center for Advanced Studies in Earth Sciences, Energy and Environment (CEACTEMA), University of Jaén, Campus Las Lagunillas s/n, 23071 Jaén, Spaind*

[d]*Institute of Chemical Process Engineering, University of Alicante, PO 99, E-3080 Alicante, Spain*

[e]*Centre for Environmental Policy, Imperial College London, Exhibition Road, London SW7 1NA, UK*

[f]*Centre for Process Systems Engineering, Imperial College London, Exhibition Road, London SW7 2AZ, UK*

†Ángel Galán-Martín and Daniel Vázquez contributed equally.

*Correspondence to: gonzalo.guillen.gosalbez@chem.ethz.ch

This document contains the supplementary information of the article "Delaying carbon dioxide removal in the European Union puts climate targets at risk", which is structured in four sections. First, the RAPID model is described. In the second section, all the data employed are presented. The third section provides some additional results. The fourth section discusses the main methodological assumptions and limitations. Finally, some supplementary references are included.

1. RAPID model

Our work explores the technical, economic, and environmental consequences of delaying CDR actions. To carry out our analysis, we developed a multi-period linear programming model named RAPID (as the acronym for RemovAl oPtImization moDel). RAPID is an energy system model focused on integrating BECCS and DACCS into the energy sector as key engineered CDR options to achieve the climate goals. In essence, RAPID identifies the most cost-effective emissions pathways by simultaneously modifying the power mix and deploying BECCS and DACCS from a particular year onwards. Although we focus on the European Union context for our analysis, RAPID could be easily extrapolated to other regions.
The mathematical formulation of RAPID is described below. First, we present the nomenclature (i.e., sets, parameters, and variables) and then describe the main equations.

1.1. Nomenclature

1.1.1. Sets

Four main sets are defined:

T := {t : Time periods of five years}
J := {j : Countries}
I := {i : Electricity generation technologies}
S := {s : DACCS technologies}
B := {b : Types of biomass}

From these main sets, we derive the following subsets:

PI_t := {t : Periods of inactivity}
GS_j := {j : Countries with geological storage capacity}
TD_i := {i : Dispatchable technologies}
RE_i := {i : Renewable technologies}
$TCCS_i$:= {i : Technologies that include carbon capture and storage}
BC_b := {b : Bioenergy crops}
BR_b := {b: Biomass residues}
BT_i := {i : Bio-based electricity technologies}

1.1.2. Parameters

The parameters employed in the model are shown in **Supplementary Table 1**.

Supplementary Table 1. Parameters used in the model

Distance parameters	Description	Units
$DC_{j,j'}$	Distance between country *j* and country *j'*.	km
$DGS_{j,j'}$	Distance between country *j* and the geological storage in country *j'*.	km
DI_j	Distance from Russia to country *j*	km
**DAC parameters**	**Description**	**Units**
$HEAT_s^{DAC}$	Heating requirements to capture 1 Gt of CO_2 for the DACCS technology *s*.	$TWh/GtCO_2$
$ELEC_s^{DAC}$	Electricity consumption to capture 1 Gt of CO_2 for DACCS technology *s*.	$TWh/GtCO_2$

HCAP	CO_2 capture efficiency for the heating system in the DACCS plant.	%
$DAC^{HEMISSIONS}$	CO_2 emissions factor for the supply of heating from natural gas in the DACCS plant.	Gt CO_2/TWh
$DAC^{NGEXTRACT}$	Life cycle CO_2 emissions intensity for the natural gas powering the DACCS plant.	Gt CO_2/TWh
DAC^{LIFE}	Expected lifetime for DACCS technology *s*.	Time periods
**Cost parameters***	**Description**	**Units**
$CAPEX_{s,t}^{DAC}$	Capital cost of the DACCS configuration *s* in period *t*.	B€/(Gt/yr)
$OPEX_{s,t}^{DAC}$	Operating cost of DACCS configuration *s* in period *t*.	B€/GtCO_2
$COST_j^{Heat}$	Cost of natural gas heating in country *j*.	B€/TWh
$CAPEX_{i,t}$	Capital cost of electricity technology *i* in period *t*.	B€/TW
$OPEX_{i,t}^{Fix}$	Fixed operating cost of electricity technology *i* in period *t*.	B€/TW
$OPEX_{i,t}^{Var}$	Variable operating cost of electricity technology *i* in period *t*.	B€/TWh
FC_b^{Bio}	Fuel costs of biomass type *b*.	B€/Gt(db)
FC_i^{Fuel}	Fuel costs of coal, natural gas, and uranium.	B€/TWh
$COST^{Pipetrans}$	Cost for transporting natural gas and CO_2 via pipeline.	B€/Gt/km
$COST^{BTrans}$	Biomass transport cost.	B€/Gt/km (db)
$COST^{Inject}$	CO_2 injection cost.	B€/Gt
$CRF_{j,i}$	Capital recovery factor for technology *i* in country *j*.	-
CRF^{DAC}	Capital recovery factor for DACCS.	-
IF	Inflation factor.	-
**CO_2 emission parameters[#]**	**Description**	**Units**
$EM_{j,i}^{Elec}$	Life cycle CO_2 emission intensity of electricity technology *i* in country *j*.	Gt CO_2/TWh
EM^{Sto}	Life cycle CO_2 emission intensity of the CO_2 transportation and storage.	Gt CO_2/Gt/km
EM^{NGT}	Life cycle CO_2 emission intensity for natural gas transportation.	Gt CO_2/Gt/km
$EM_{j,b}^{BP}$	CO_2 emission intensity for the cultivation of biomass type *b* in country *j*.	Gt CO_2/Gt (wb)
EM_b^{BBio}	Direct emissions from burning pellets of type *b* in a Biomass w/o CCS plant.	Gt CO_2/Gt (db)
EM_b^{BBECCS}	Direct emissions from burning pellets of type *b* in a BECCS plant.	Gt CO_2/Gt (db)
EM^{BPe}	Life cycle CO_2 emission intensity of drying and pelletizing biomass.	Gt CO_2/Gt (db)
EM^{BT}	Life cycle CO_2 emission intensity of biomass pellets transportation.	Gt CO_2/Gt/km (wb)
**Biomass parameters[#]**	**Description**	**Units**
CC_b	Carbon content of biomass type *b*.	kg/kg (wb)
HUM_b	Water content of biomass *b*.	kg/kg (wb)

$\mathrm{BVAL}_b^{\mathrm{Biomass}}$	Electricity conversion efficiency of Biomass power plants w/o CCS per type of biomass *b*.	TWh/Gt (db)
$\mathrm{BVAL}_b^{\mathrm{BECCS}}$	Electricity conversion efficiency of BECCS plants per type of biomass *b*.	TWh/Gt (db)
BREM_b	CO_2 removed with biomass type *b*.	$GtCO_2$/Gt (db)
$\mathrm{PY}_{j,b}$	Yield of the biomass deployed by biomass type *b* in country *j* in one period.	Gt/Mha/period (db)
$\mathrm{LOSS}^{\mathrm{Cul}}$	Biomass losses due to poor harvest practices, inappropriate harvest technology, and inadequate scheduling.	%
$\mathrm{LOSS}^{\mathrm{Pell}}$	Biomass losses at the pelleting plant due to inadequate handling and poor storage conditions.	%
**Storage parameters**	**Description**	**Units**
$\mathrm{STO}_{j,i}^{\mathrm{Elec}}$	CO_2 post-combustion captured in fossil-fuel electricity technology *i* with CCS in country *j* (only Coal CCS and NG CCS).	$GtCO_2$/TWh
$\mathrm{STO}_i^{\mathrm{B}}$	CO_2 post-combustion captured with bio-based electricity technology *i*.	$GtCO_2$/Gt (db)
$\mathrm{STO}_j^{\mathrm{Cap}}$	CO_2 geological storage capacity in country *j*.	$GtCO_2$
**Demand parameters**	**Description**	**Units**
$\mathrm{D}_{j,t}^{\mathrm{Elec}}$	Electricity demand in country *j* in period *t*.	TWh
**Limit parameters**	**Description**	**Units**
$\mathrm{LIM}_j^{\mathrm{Heat}}$	Upper bound on the heat generated in country *j*.	TWh/yr
$\mathrm{GEN}_{j,i}^{\mathrm{Pot}}$	Upper bound on the electricity generated with technology *i* in country *j*.	TWh/yr
$\mathrm{LIM}_j^{\mathrm{Coal}}$	Limit on the capacity of technologies that use coal in country *j.*	TW
$\mathrm{LIM}_j^{\mathrm{NG}}$	Limit on the capacity of technologies that use natural gas in country *j*.	TW
$\mathrm{LIM}_j^{\mathrm{Nuclear}}$	Limit on the nuclear power capacity in country *j*.	TW
$\mathrm{LIM}_j^{\mathrm{Area}}$	Area of marginal land available for energy crops cultivation in country *j*.	Mha
$\mathrm{LIM}_{j,b}^{\mathrm{BR}}$	Availability of biomass residues of type *b* in country *j* in one period.	Gt/period (wb)
**Capacity today parameters**	**Description**	**Units**
$\mathrm{CAP}_{j,i}^{\mathrm{Today}}$	Current capacity installed of technology *i* in country *j*.	TW
$\mathrm{PARCAP}_{i,t}^{\mathrm{Today}}$	Binary parameter (0 if today's capacity of technology *i* is still active in period *t*, 0 otherwise)	-
**Other parameters**	**Description**	**Units**
BUC	Backup coefficient denoting the minimum capacity of dispatchable technologies required to compensate each MW of intermittent technologies.	-
$\mathrm{CF}_{i,t}$	Capacity factor of technology *i* in period *t*.	-
DPER	Duration of a period.	y

YH	Hours in a year.	h/yr
$\mathrm{UL_i}$	Useful life of technology *i*.	period
$\mathrm{E^{Loss}}$	Electricity transmissions losses.	%/km
$\mathrm{CAP^{EF}}$	Maximum diffusion rate of technologies	-
$\mathrm{HHV^{NG}}$	Higher heating value of natural gas	MJ/kg
$\mathrm{INITIAL^{DAC}}$	Maximum initial DAC capacity that can be installed.	Gt/yr
$\mathrm{INITIAL^{BECCS}}$	Maximum initial BECCS capacity that can be installed.	TW
$\mathrm{OPEN^{Tech}}$	Maximum power generation capacity that can be expanded in Europe every period for each energy generation technology – except BECCS – in addition to the assumed exponential growth.	TW

*B€ stands for Billion euros, which corresponds to 10^9 euros.

#The biomass parameters refer to either wet or dry basis, i.e., wb and db, respectively.

1.1.3. **Variables**

The variables used in the model are shown in **Supplementary Table 2**.

***Supplementary Table 2*.** Variables used in the model.

**Removal variables**	**Description**	**Units**
$rr_{j,t,s}^{DAC}$	Amount of CO_2 removed from the atmosphere by DACCS in country *j* and period *t* and with technology *s*.	$GtCO_2$
$r_{j,t}^{Total}$	Total amount of CO_2 removed from the atmosphere via photosynthesis (BECCS and biomass) or through chemical reactions (DACCS) in country *j* in period *t*.	$GtCO_2$
**Total emissions and cost variables**	**Description**	**Units**
$c_{j,t}^{Country}$	Total cost of country *j* in period *t*.	B€
$e_{j,t}^{Country}$	Total emissions of country *j* in period *t*.	$GtCO_2$
$e_{j,t}^{Power}$	Emissions from the power sector (except BECCS) in country *j* and period *t.*	$GtCO_2$
$e_{j,t}^{DACCS}$	Emissions from the DACCS system in country *j* and period *t.*	$GtCO_2$
$e_{j,t}^{BT}$	Emissions from BECCS and Biomass in country *j* and period *t.*	$GtCO_2$
$e_{j,t}^{ht}$	Emissions associated with the natural gas transportation for heating in country *j* and period *t.*	$GtCO_2$
$e_{j,t}^{CO2t}$	Emissions associated with the CO_2 transportation and injection in geological sites in country *j* and period *t.*	$GtCO_2$
$c_{j,t}^{Power}$	Costs from the power sector (except BECCS) in country *j* and period *t.*	$GtCO_2$
$c_{j,t}^{DACCS}$	Costs of DACCS in country *j* and period *t.*	$GtCO_2$

$c_{j,t}^{BT}$	Costs of BECCS and Biomass in country *j* and period *t.*	$GtCO_2$
$c_{j,t}^{ht}$	Costs associated with the natural gas transportation from Russia (for heating) in country *j* and period *t.*	$GtCO_2$
$c_{j,t}^{CO2t}$	Costs associated with the CO_2 transportation and injection in geological sites in country *j* and period *t.*	$GtCO_2$
**Electricity generation and capacity variables**	**Description**	**Units**
$gen_{j,t}^{Total}$	Amount of electricity generated in country *j* in period *t*.	TWh
$gen_{j,i,t}^{Tech}$	Amount of electricity generated in country *j* using technology *i* in period *t.*	TWh
$gen_{j,i,t}^{ST}$	Amount of standard electricity produced in country *j* with technology *i* in period *t*.	TWh
$gen_{j,i,t}^{BU}$	Amount of backup electricity produced in country *j* using technology *i* in period *t*.	TWh
$cap_{j,i,t}^{Avail}$	Capacity available in country *j* of technology *i* in period *t*.	TW
$cap_{j,i,t}^{Exp}$	Expansion in capacity in country *j* with technology *i* in period *t*.	TW
$cap_{j,i,t}^{ST}$	Standard power capacity in country *j* of a technology *i* in period *t*.	TW
$cap_{j,i,t}^{BU}$	Backup power capacity in country *j* of a technology *i* in period *t*.	TW
$dac_{j,s,t}^{Avail}$	Removal capacity with DACCS in country *j* in period *t*.	$GtCO_2$/yr
$dac_{j,s,t}^{Exp}$	Expansion in DACCS capacity in country *j* in period *t*.	$GtCO_2$/yr
**Heating generation variables**	**Description**	**Units**
$heat_{j,t}^{Gen}$	Heating from natural gas produced in country *j* in period *t*.	TWh
$import_{j,t}^{Russia}$	Natural gas for heating imported from Russia in country *j* and period *t.*	TWh
**Transport variables**	**Description**	**Units**
$gen_{j,j',t}^{Trans}$	Electricity traded from country *j* to country *j'* in period *t*.	TWh
$heat_{j,j',t}^{Trans}$	Heating transported from country *j* to country *j'* in period *t*.	TWh
$r_{j,j',t}^{Sto}$	Amount of CO_2 transported from country *j* and stored in *j'* in period *t*.	$GtCO_2$
**Area variables**	**Description**	**Units**
$area_{j,b,t}$	Area dedicated to grow bioenergy crop *b* in country *j* in period *t*.	Mha
**Biomass variables**	**Description**	**Units**
$wb_{j,b,t}$	Wet biomass produced in country *j* with biomass type *b* in period *t*.	Gt (wb)
$bb_{j,b,t}^{Total}$	Total pellets of biomass type *b* combusted in country *j* in period *t*.	Gt (db)

$bb_{j,b,t}^{Biomass}$	Amount of pellets of biomass type *b* combusted in Biomass w/o CCS plants in country *j* in period *t*.	Gt (db)
$bb_{j,b,t}^{BECCS}$	Amount of pellets of biomass type *b* combusted in BECCS plants in country *j* in period *t*.	Gt (db)
$db_{j,b,t}$	Dry biomass (pellets) produced in country *j* with biomass type *b* in period *t*.	Gt (db)
$tb_{j,j',b,t}$	Amount of biomass of type *b* transported from *j* to *j'* in period *t*.	Gt (db)
**Objective variables**	**Description**	**Units**
obj^{Env}	Environmental objective. CO_2 emissions balance.	$GtCO_2$
obj^{Eco}	Economic objective. Total costs.	B€

1.2. The RAPID model: mathematical formulation

RAPID takes the form of a linear programming (LP) model, which was implemented in the algebraic modeling system GAMS[1] version 32.2.0. RAPID features in total 305,314 continuous variables and 109,068 equations and can be solved using standard LP solvers. We next describe the main equations of RAPID, organized into five main blocks: load-meeting and operational constraints, emissions-related equations, costs equations, inactivity equations, and objective function-related equations. Variables are written in italics, while parameters are given in capital letters.

1.2.1. Load-meeting and operations constraints

These constraints model the design, expansion, and operation of the power system, as well as the generation and transmission of electricity between production and load regions.

The first equation (Eq. 1) computes the total electricity generated in country *j* in a particular period *t* ($gen_{j,t}^{Total}$) from the amount of electricity produced by each power technology *i* in each country *j* in period *t* ($gen_{j,i,t}^{Tech}$).

$$gen_{j,t}^{Total} = \sum_{i \in I} gen_{j,i,t}^{Tech} \qquad \forall j \in J, t \in T \qquad \textit{Eq. 1}$$

Note that the electricity generated can be used for standard consumption or to provide a flexible backup to handle the intermittency of renewables and ensure the system's reliability. The relationship between dispatchable and non-dispatchable power technologies is explained later in this document (Eq. 10). Notably, the electricity generated ($gen_{j,i,t}^{Tech}$) is modeled as the summation of two terms, the standard, and backup generation ($gen_{j,i,t}^{ST}$ and $gen_{j,i,t}^{BU}$, respectively), as shown in Eq. 2.

$$gen_{j,i,t}^{Tech} = gen_{j,i,t}^{ST} + gen_{j,i,t}^{BU} \qquad \forall j \in J, i \in I, t \in T \qquad \textit{Eq. 2}$$

The amount of electricity generated is linked to the installed capacities through the capacity factor parameter ($\mathrm{CF_i}$) and the annual operating hours (YH) within each period (Eq. 3 and 4). Regarding the standard electricity generation and capacity (represented by variables $cap_{j,i,t}^{ST}$ and $gen_{j,i,t}^{ST}$, respectively in Eq. 3), we note that generation sources might sometimes operate below their maximum capacity; consequently, Eq. 3 is imposed as an inequality. Conversely, for the

backup systems, the capacity installed ($cap_{j,i,t}^{BU}$) must always be active to ensure the system's reliability as the backstop for the intermittency of the renewable (i.e., Eq. 4 is defined as an equality constraint).

$$gen_{j,i,t}^{ST} \leq cap_{j,i,t}^{ST} \cdot \mathrm{YH} \cdot \mathrm{DPER} \cdot \mathrm{CF_i} \qquad \forall j \in J, i \in I, t \in T \qquad \textit{Eq. 3}$$
$$gen_{j,i,t}^{BU} = cap_{j,i,t}^{BU} \cdot \mathrm{YH} \cdot \mathrm{DPER} \cdot \mathrm{CF_i} \qquad \forall j \in J, i \in I, t \in T \qquad \textit{Eq. 4}$$

Eq. 5 ensures that, for each period *t*, the domestic electricity generated in each country *j*, plus the power flows imported from countries *j'* to country *j*, minus the exported power from country *j* to countries *j'* must be enough to fulfill the electricity demand. Note that the electricity demand in each country *j* in each period *t* is given by both the standard electricity demand ($\mathrm{D_{j,t}^{ELEC}}$) plus the energy needed by the DACCS facilities *s* deployed in *j*. The latter term is estimated from the amount of CO_2 removed in country *j* and period *t* with all types *s* of DACCS (provided by the variable $rr_{j,t,s}^{DAC}$) and the associated electricity requirements ($\mathrm{ELEC_s^{DAC}}$).

$$\begin{aligned} & gen_{j,t}^{Total} \\ & + \sum_{j' \in J} gen_{j',j,t}^{Trans}\left[1 - \mathrm{E}^{Loss} \cdot \mathrm{DC_{j',j}}\right] - \sum_{j' \in J} gen_{j,j',t}^{Trans} \\ & \qquad \geq \mathrm{D_{j,t}^{ELEC}} + \sum_{s \in S} rr_{j,t,s}^{DAC} \mathrm{ELEC_s^{DAC}} \qquad \forall j \in J, t \in T \end{aligned} \qquad \textit{Eq.5}$$

Additionally, Equation 6 limits the energy dependency on foreign energy suppliers. Accordingly, Eq. 6 forces that at least a certain percentage of the total electricity demand in a country *j* must be met with electricity generated domestically (e.g., 50%, parameter ED).

$$gen_{j,t}^{Total} - \sum_{j' \in J} gen_{j,j',t}^{Trans} \geq \mathrm{ED}\left(\sum_{s \in S} rr_{j,t,s}^{DAC} \mathrm{ELEC_s^{DAC}} + \mathrm{D_{j,t}^{ELEC}}\right) \qquad \textit{Eq. 6}$$
$$\forall j \in J, t \in T$$

Eq. 7 computes the capacity available for power technology *i* in country *j* in period *t* ($cap_{j,i,t}^{Avail}$) from the capacity available today ($\mathrm{CAP_{j,i}^{Today}}$) plus the capacity expansions ($cap_{j,i,t}^{Exp}$) taking place in time periods before *t*, in both cases considering their corresponding useful life (modeled via parameters $\mathrm{PARCAP_{i,t}^{Today}}$ and $\mathrm{UL_i}$). Binary parameter $\mathrm{PARCAP_{i,t}^{Today}}$ in Eq.7 takes a value of one if today's capacity of *i* remains open in period *t* (details in Eq. 48 and Eq. 49), and it is zero otherwise.

$$cap_{j,i,t}^{Avail} = \mathrm{PARCAP_{i,t}^{Today}} \mathrm{CAP_{j,i}^{Today}} + \sum_{t'=t-\mathrm{UL_i}+1}^{t'=t} cap_{j,i,t'}^{Exp} \qquad \textit{Eq. 7}$$
$$\forall j \in J, i \in I, t \in T$$

Similarly, as with the generation (Eq. 2), the total capacity available of technology *i* in country *j* and period *t* is given by the summation of the standard and backup capacities (Eq. 8). The backup capacity of the intermittent renewable technologies (not belonging to the subset *TD* of dispatchable technologies) is zero, as they cannot act as a backup (Eq. 9).

$$cap_{j,i,t}^{Avail} = cap_{j,i,t}^{ST} + cap_{j,i,t}^{BU} \qquad \forall j \in J, i \in I, t \in T \qquad \textit{Eq. 8}$$

$$cap^{BU}_{j,i,t} = 0 \qquad \forall j \in J, i \notin TD, t \in T \qquad \textit{Eq. 9}$$

Eq. 10 ensures the system's reliability by enforcing that the load demand is met at any time. Under unfavorable weather conditions, the capacity available with intermittent renewable technologies (i.e., wind onshore, wind offshore, solar PV open-ground, and solar PV rooftop installation) is always supported by ancillary systems provided by the firm and dispatchable technologies (i.e., the subset of technologies TD). The ratio between dispatchable and non-dispatchable technologies is modeled through the backup coefficient (BUC), which ensures that the backup is higher than a percentage of the standard capacity of the non-dispatchable technologies (e.g., 0.5), as shown by Eq. 10.

$$\sum_{i \in TD_i} cap^{BU}_{j,i,t} \geq \mathrm{BUC} \sum_{i \notin TD_i} cap^{ST}_{j,i,t} \qquad \forall j \in J, t \in T \qquad \textit{Eq. 10}$$

The following equations impose limits to the capacity installed with each technology *i* in each country *j* and period *t*. Eq. 11 and 12 apply to the fossil-based power technologies, i.e., coal power w/ and w/o CCS and natural gas power w/ and w/o CCS, respectively, which compete for the same resources (i.e., coal and natural gas). These equations impose limits on electricity generation based on the maximum installed capacity allowed in each country, defined by parameters $\mathrm{LIM}_j^{\mathrm{Coal}}$and $\mathrm{LIM}_j^{\mathrm{NG}}$ respectively.

$$cap^{Avail}_{j,Coal,t} + cap^{Avail}_{j,CoalCCS,t} \leq \mathrm{LIM}_j^{\mathrm{Coal}} \qquad \forall j \in J, t \in T \qquad \textit{Eq. 11}$$

$$cap^{Avail}_{j,Ngas,t} + cap^{Avail}_{j,NgasCCS,t} \leq \mathrm{LIM}_j^{\mathrm{NG}} \qquad \forall j \in J, t \in T \qquad \textit{Eq. 12}$$

Similarly, for nuclear power, Eq. 13 enforces that the capacity installed in each country *j* and period *t* cannot exceed a given limit defined by parameter $\mathrm{LIM}_j^{\mathrm{Nuclear}}$.

$$cap^{Avail}_{j,nuclear,t} \leq \mathrm{LIM}_j^{\mathrm{Nuclear}} \qquad \forall j \in J, t \in T \qquad \textit{Eq. 13}$$

Eq. 14 applies to renewable technologies, excluding bio-based technologies (i.e., wind, geothermal, hydropower, and solar, **Supplementary Table 4**). The equation constrains the total amount of electricity generated in country *j* with renewable technology *i* (set of renewable technologies RE_i), given by the summation of both the standard and back-up generation ($gen^{Tech}_{j,i,t}$ in Eq.2), based on the availability of the corresponding renewable resource in country *j* and period *t* ($\mathrm{GEN}_{j,i}^{\mathrm{Pot}}$). Note that for intermittent wind and solar PV, which are not included in the subset of dispatchable technologies TD, the back-up capacity is set to zero in Eq. 9 and, therefore, only standard generation is considered.

$$gen^{Tech}_{j,i,t} \leq \mathrm{GEN}_{j,i}^{\mathrm{Pot}}\mathrm{DPER} \qquad \forall j \in J, i \in RE_i, t \in T \qquad \textit{Eq. 14}$$

For the biomass-based technologies, i.e., BECCS and Biomass w/o CCS power, the maximum generation is given by the biomass resources availability. In the case of bioenergy crops, the limit can be defined from the marginal land available to grow crops and, in the case of residues, from the amount of agricultural and forestry residues available from industrial activities. Hence, the

electricity generated with biomass-based electricity technologies is limited by the availability of pellets that can be produced from each type of biomass *b*.

Eq. 15 and Eq. 16 provide the electricity generated with biomass (w/o CCS) and BECCS, respectively, where $bb_{j,b,t}^{Biomass}$ and $bb_{j,b,t}^{BECCS}$ denote the mass of dry biomass (in the form of pellets) of type *b* burned in Biomass w/o CCS and BECCS power plants in country *j* and period *t*, respectively. Note that the amount of pellets that can be produced is ultimately constrained by the availability of marginal land and biomass residues (Eq. 21 and Eq. 22, respectively). $\mathrm{BVAL_b^{Biomass}}$ and $\mathrm{BVAL_b^{BECCS}}$ are parameters representing the yield of biomass conversion into electricity in biomass plants w or w/o CCS (expressed in TWh per Gt of biomass measured on a dry basis). Note that for the case of BECCS, parameter $\mathrm{BVAL_b^{BECCS}}$ considers the energy penalty linked to the CCS system (calculations in section 2.2.5, Eqs. 59 to 62).

$$gen_{j,i,t}^{Tech} = \sum_{b \in B} bb_{j,b,t}^{Biomass} \, \mathrm{BVAL_b^{Biomass}} \qquad \forall j \in J, i = Biomass, t \in T \qquad \textit{Eq. 15}$$

$$gen_{j,i,t}^{Tech} = \sum_{b \in B} bb_{j,b,t}^{BECCS} \mathrm{BVAL_b^{BECCS}} \qquad \forall j \in J, i = BECCS, t \in T \qquad \textit{Eq. 16}$$

The total amount of pellets of biomass type *b* combusted in country *j* and period *t* ($bb_{j,b,t}^{Total}$) is given by the summation of the pellets consumed by the Biomass w/o CCS ($bb_{j,b,t}^{Biomass}$) and BECCS ($bb_{j,b,t}^{BECCS}$) plants, as shown in Eq. 17:

$$bb_{j,b,t}^{Total} = bb_{j,b,t}^{Biomass} + bb_{j,b,t}^{BECCS} \qquad \forall j \in J, b \in B, t \in \qquad \textit{Eq. 17}$$

The total mass of biomass pellets of type *b* available to be burned in country *j* and period *t* ($bb_{j,b,t}^{Total}$) is given by the domestic biomass pellets used ($db_{j,b,t}$ measured on a dry basis) plus the imports of pellets of type *b* imported from countries *j'* (to country *j*) minus the exports of pellets of type *b* from country *j* (to countries *j'*), as shown in Eq. 18.

$$bb_{j,b,t}^{Total} = db_{j,b,t} + \sum_{j' \in J} tb_{j',j,b,t} - \sum_{j' \in J} tb_{j,j',b,t} \qquad \forall j \in J, b \in B, t \in T \qquad \textit{Eq. 18}$$

The pelletizing process consists of four main stages: pre-treatment of the raw biomass, drying, conditioning, and pellets manufacturing. In essence, the biomass is converted from wet raw material to dry biomass pellets (densified biomass). Hence, Eq. 19 establishes the relationship between the dry ($db_{j,b,t}$) and wet weight biomass ($wb_{j,b,t}$) considering the moisture content of each biomass type *b* ($\mathrm{HUM_b}$) as well as the losses during the pelleting stage due to, for example, inadequate handling of biomass resources or poor storage conditions ($\mathrm{LOSS^{Pell}}$, expressed as a percentage).

$$db_{j,b,t} = wb_{j,b,t}(1 - \mathrm{HUM_b})(1 - \mathrm{LOSS^{Pell}}) \qquad \forall j \in J, b \in B, t \in T \qquad \textit{Eq. 19}$$

Two types of second-generation biomass feedstocks are considered, i.e., dedicated bioenergy crops and biomass residues. Therefore, the amount of biomass feedstock *b* available in a country *j* in period *t* (either on a wet or dry basis) is given by the energy crops cultivated on marginal land and the residues available.

For the bioenergy crops (subset BC_b), the amount of biomass growth in each country *j* and period t ($db_{j,b,t}$) is calculated, as shown in Eq. 20, from the marginal land devoted to each particular crop ($area_{j,b,t}$) and the production yield parameter ($\mathrm{PY_{j,b}}$). Note that we also consider biomass losses in the cultivation phase of the bioenergy crops ($\mathrm{LOSS^{Cul}}$, expressed as a percentage), which may arise due to poor harvest practices, inappropriate harvest technologies, or inadequate scheduling and timing of the agricultural activities.

$$wb_{j,b,t}(1-\mathrm{HUM_b}) = area_{j,b,t}\mathrm{PY_{j,b}}(1-\mathrm{LOSS^{Cul}}) \qquad \forall j \in J, b \in BC_b, t \in T \qquad \textit{Eq. 20}$$

The land area used for growing bioenergy crops in each country *j* and period *t* is constrained by the marginal land available in the country ($\mathrm{LIM_j^{Area}}$) as in Eq. 21.

$$\sum_{b \in BC_b} area_{j,b,t} \le \mathrm{LIM_j^{Area}} \qquad \forall j \in J, t \in T \qquad \textit{Eq. 21}$$

Concerning the biomass residues (subset BR_b), the mass of wet biomass residues of type *b* used in country *j* and period *t* is limited by its availability in that country ($\mathrm{LIM_{j,b}^{BR}}$), as shown in Eq. 22.

$$wb_{j,b,t} \le \mathrm{LIM_{j,b}^{BR}} \qquad \forall j \in J, b \in BR_b, t \in T \qquad \textit{Eq. 22}$$

Finally, Eq. 23 prevents countries from behaving as intermediate traders in biomass markets by forcing the maximum amount of pellets exported from *j* to *j'* ($\sum_{j' \in J} tb_{j,j',b,t}$) to be lower than the biomass produced in the same country *j* for every period *t* ($db_{j,b,t}$).

$$db_{j,b,t} - \sum_{j' \in J} tb_{j,j',b,t} \ge 0 \qquad \forall j \in J, b \in B, t \in T \qquad \textit{Eq. 23}$$

The previous equations impose limits on the total capacities installed and the electricity provided based on the resources available (e.g., wind resource, land). However, other factors limit the diffusion of existing and new technologies, ultimately constraining their maximum deployment rate. For instance, the speed of deployment may be affected by market forces, competition, the adaptation of new infrastructure, learning rates, or social acceptance issues, among others[2]. Accordingly, we introduced in the model a capacity expansion factor ($\mathrm{CAP^{EF}}$) that imposes a maximum growth rate relative to previous periods.

Eq. 24 applies to the initial period ($\mathrm{t} = 1$, e.g., 2020), which considers the initial installed capacity (i.e., capacity in 2019, parameter $\mathrm{CAP_{j,i}^{Today}}$) plus the expansion in capacity taking place in that year. Additionally, parameter $\mathrm{OPEN^{Tech}}$ ensures that the technologies not deployed today (e.g., fossil fuels + CCS) could still be implemented in the future by assuming that a minimum capacity is already installed. Eq. 25 applies from the initial period onwards. $\mathrm{CAP^{EF}}$ represents the maximum annual growth rate (e.g., 20%), while DPER considers the length of the period (i.e., five years).

$$\sum_{j \in J} cap_{j,i,t}^{Avail} \le \sum_{j \in J} \mathrm{CAP_{j,i}^{Today}}\left(1+\mathrm{CAP^{EF}}\right) + \mathrm{OPEN^{Tech}} \qquad \forall i \ne BECCS, t = 1 \qquad \textit{Eq. 24}$$

$$\sum_{j\in J} cap_{j,i,t}^{Avail} \leq \sum_{j\in J} cap_{j,i,t-1}^{Avail} \left(1 + \mathrm{CAP^{EF}}\right)^{\mathrm{DPER}} + \mathrm{OPEN^{Tech}} \quad \forall i \neq BECCS, t > 1 \qquad \textit{Eq. 25}$$

Concerning BECCS and DACCS, their maximum diffusion rate is modeled using Eqs. 26-29, where we consider an initial installed capacity for DACCS and BECCS (parameters $\mathrm{INITIAL^{DAC}}$ and $\mathrm{INITIAL^{BECCS}}$, respectively in Eq. 26 and 28). Moreover, to model the consequences of inaction on CDR, we assume that the deployment of DACCS and BECCS starts in the first non-inactive period. The periods of inactivity are selected manually to control the delay in the CDR actions. Eqs. 26 and 27 correspond to the capacity expansion of DACCS, and Eqs. 28 and 29 apply to BECCS. Note that to explore the implications of inaction on DACCS and BECCS, we fix their capacity to zero during the inactive periods, as explained later in the document (Eqs. 49 and 50).

$$\sum_{j\in J}\sum_{s\in S} dac_{j,s,t}^{Avail} \leq \mathrm{INITIAL^{DAC}} \quad \forall t = |PI| + 1 \qquad \textit{Eq. 26}$$

$$\sum_{j\in J}\sum_{s\in S} dac_{j,s,t}^{Avail} \leq \sum_{j\in J}\sum_{s\in S} dac_{j,s,t-1}^{Avail} \left(1 + \mathrm{CAP^{EF}}\right)^{\mathrm{DPER}} \quad \forall t > |PI| + 1 \qquad \textit{Eq. 27}$$

$$\sum_{j\in J} cap_{j,i,t}^{Avail} \leq \mathrm{INITIAL^{BECCS}} \quad \forall i = BECCS, t = |PI| + 1 \qquad \textit{Eq. 28}$$

$$\sum_{j\in J} cap_{j,i,t}^{Avail} \leq \sum_{j\in J} cap_{j,i,t-1}^{Avail} \left(1 + \mathrm{CAP^{EF}}\right)^{\mathrm{DPER}} \quad \forall i = BECCS, t > |PI| + 1 \qquad \textit{Eq. 29}$$

Besides the power needs, RAPID also considers the heating requirements for the DACCS technologies, covered by natural gas. Hence, Eq. 30 defines the natural gas balance for every period t considering that the amount of heating produced in a country j ($heat_{j,t}^{Gen}$), plus the amount imported from countries j' to country j ($\sum_{j'\in J} heat_{j',j,t}^{Trans}$), minus the amount exported to other countries j' ($\sum_{j'\in J} heat_{j,j',t}^{Trans}$), must equal the demand. The heating demand of DACCS is computed from the amount of CO_2 removed from the atmosphere with all the configurations s ($rr_{j,t,s}^{DAC}$) and their heating requirements ($\mathrm{HEAT_s^{DAC}}$, expressed in TWh per Gt of CO_2 removed).

$$heat_{j,t}^{Gen} + \sum_{j'\in J} heat_{j',j,t}^{Trans} - \sum_{j'\in J} heat_{j,j',t}^{Trans} + import_{j,t}^{Rusia} = \sum_{s\in S} rr_{j,t,s}^{DAC}\mathrm{HEAT_s^{DAC}} \quad \forall j \in J, t \in T \qquad \textit{Eq. 30}$$

Finally, Eq. 31 imposes that the heating provided in each country j in each period t ($heat_{j,t}^{Gen}$) should not exceed the natural gas heating resources available in that country ($\mathrm{LIM_j^{Heat}}$). Note that we consider that natural gas can be imported from Russia, assuming an unlimited supply.

$$heat_{j,t}^{Gen} \leq \mathrm{LIM_j^{Heat}} \quad \forall j \in J, t \in T \qquad \textit{Eq. 31}$$

1.2.2. Emission-related equations

These equations model the CO_2 balance, i.e., the life cycle emissions accounting, including the CO_2 capture, transportation, and storage. The CO_2 emissions balance accounts for both the positive emissions (life cycle emissions emitted to the atmosphere) and the negative ones (removals from the atmosphere via BECCS and DACCS).

The total positive life cycle emissions in each country *j* and period *t* ($e_{j,t}^{Country}$) are computed in Eq. 32 as the summation of the emissions associated with the following terms: electricity generation ($e_{j,t}^{Power}$), excluding those emissions linked to the biomass-based power technologies for which a tailored balance is performed, the operation of the DACCS facilities ($e_{j,t}^{DACCS}$) and the biomass-based technologies ($e_{j,t}^{BT}$), the natural gas transportation ($e_{j,t}^{ht}$) and the CO_2 transportation and injection in geological sites ($e_{j,t}^{CO2t}$).

$$e_{j,t}^{Country} = e_{j,t}^{Power} + e_{j,t}^{DACCS} + e_{j,t}^{BT} + e_{j,t}^{ht} + e_{j,t}^{CO2t} \qquad \forall j \in J, t \in T \qquad \textit{Eq. 32}$$

Eq. 33 computes the positive emissions of electricity generation for all the technologies except for the biomass-based ones (i.e., all *i* that do not belong to the subset *BT*) from the electricity generated ($gen_{j,i,t}^{Tech}$) and the life cycle emissions intensity ($\mathrm{EM}_{\mathrm{j,i}}^{\mathrm{Elec}}$).

$$e_{j,t}^{Power} = \sum_{i \notin BT_i} gen_{j,i,t}^{Tech} \mathrm{EM}_{\mathrm{j,i}}^{\mathrm{Elec}} \qquad \forall j \in J, t \in \mathrm{T} \qquad \textit{Eq. 33}$$

The positive emissions attributed to the DACCS facilities installed in each country *j* and period *t* ($e_{j,t}^{DACCS}$) are computed with Eq. 34 from the emissions related to natural gas extraction and the direct emissions from natural gas combustion not captured in the DACCS facility (determined from the total emissions $\mathrm{DAC^{HEMISSIONS}}$, considering the heating requirements, $\mathrm{HEAT_s^{DAC}}$, and a specific capture efficiency value, HCAP, e.g., 90%).

$$\begin{aligned} e_{j,t}^{DACCS} = & \sum_{s \in S} rr_{j,t,s}^{DAC} \mathrm{HEAT_s^{DAC}}\, \mathrm{DAC^{NGEXTRACT}} \\ & + \sum_{s \in S} rr_{j,t,s}^{DAC} \mathrm{HEAT_s^{DAC}} \mathrm{DAC^{HEMISSIONS}} (1 - \mathrm{HCAP}) \\ & \forall j \in J, t \in T \end{aligned} \qquad \textit{Eq. 34}$$

The positive emissions from the biomass-based technologies in each country *j* and period *t* are linked to their supply chain activities (Eq. 35). First, the emissions during the production/cultivation phase of biomass type *b* are computed considering the amount of wet biomass produced ($wb_{j,b,t}$), together with the emission intensity associated with the crop production ($\mathrm{EM_{j,b}^{BP}}$). Second, the emissions of biomass conversion into pellets are obtained from the emissions intensity of the pelletizing step ($\mathrm{EM^{BPe}}$) and the biomass processed. The pellets can be used domestically (in the same country) or transported abroad. The amount of pellets of type *b* consumed within a country is, hence, provided by its domestic production ($db_{j,b,t}$) minus the exports to other countries *j'* ($tb_{j,j',b,t}$). The emissions due to local transportation are computed considering a constant internal distance from the pelleting to the power plants ($\mathrm{DC_{j,j}}$) and a given emissions intensity for road transportation via trucks ($\mathrm{EM^{BT}}$). Finally, the emissions balance considers also the direct emissions at the bio-based power plants (Biomass w/o CCS and

BECCS), computed from the mass of pellets burnt ($bb_{j,b,t}^{Biomass}$and $bb_{j,b,t}^{BECCS}$) and the post-combustion direct emissions at the plant ($\mathrm{EM_b^{BBio}}$ and $\mathrm{EM_b^{BBECCS}}$).

$$\begin{aligned} e_{j,t}^{BT} &= \sum_{b\in B} wb_{j,b,t}\mathrm{EM_{j,b}^{BP}} + \sum_{b\in B} db_{j,b,t}\mathrm{EM^{BPe}} \\ &\quad + \sum_{b\in B}\left(db_{j,b,t} - \sum_{j'\in J} tb_{j,j',b,t}\right)\mathrm{DC_{j,j}EM^{BT}} \\ &\quad + \sum_{j'\in J}\sum_{b\in B} tb_{j',j,b,t}\mathrm{DC_{j',j}EM^{BT}} + \sum_{b\in B} bb_{j,b,t}^{Biomass}\,\mathrm{EM_b^{BBio}} + \sum_{b\in B} bb_{j,b,t}^{BECCS}\,\mathrm{EM_b^{BBECCS}} \\ &\quad \forall j\in J, t\in T \end{aligned} \qquad \textit{Eq. 35}$$

Eq. 36 determines the emissions associated with the transportation of natural gas to cover the heating needs of DACCS. These are calculated from the amount of natural gas imported from Russia ($import_{j,t}^{Russia}$), estimated considering the natural gas higher heating value ($\mathrm{HHV^{NG}}$), the distance between countries ($\mathrm{DI_j}$) and the emission intensity associated with transportation via pipelines ($\mathrm{EM^{NGT}}$). Note that natural gas power technologies (w/ or w/o CCS included in the subset *NG*) also consume natural gas as feedstock; however, the life cycle emissions associated with this fossil feedstock are already accounted for in the electricity generation equation (Eq.33).

$$e_{j,t}^{ht} = \frac{import_{j,t}^{Russia}}{\mathrm{HHV^{NG}}}\mathrm{DI_j EM^{NGT}} \qquad \forall j\in J, t\in T \qquad \textit{Eq. 36}$$

Finally, Eq. 37 provides the emissions associated with the transportation and injection of the captured CO_2 ($e_{j,t}^{CO2t}$). These emissions are determined from the total amount of CO_2 captured at the BECCS, DACCS, and fossil-fuel power plants with CCS ($r_{j,j',t}^{Sto}$), the CO_2 transportation distance from the capture point to the geological sites ($\mathrm{DGS_{j,j'}}$) and the emissions intensity parameter ($\mathrm{EM^{Sto}}$).

$$e_{j,t}^{CO2t} = \sum_{j'\in GS_{j'}} r_{j,j',t}^{Sto}\mathrm{DGS_{j,j'}EM^{Sto}} \qquad \forall j\in J, t\in T \qquad \textit{Eq. 37}$$

The total amount of CO_2 removed from the atmosphere ($r_{j,t}^{Total}$) is computed from Eq. 38 as the summation of the CO_2 removed from DACCS and BECCS, modeled as a negative entry in the system (minus sign in Eq. 51). Variable $rr_{j,t,s}^{DAC}$ denotes the CO_2 captured via a chemical reaction in the DACCS plants in each country *j* and period *t* and with each technology *s*. The CO_2 uptake by the biomass via photosynthesis during its growth is calculated from the biomass types *b* produced in the country ($wb_{j,b,t}$) and their CO_2 uptake per mass of biomass type *b* (parameter $\mathrm{BREM_b}$).

$$r_{j,t}^{Total} = \sum_{s\in S} rr_{j,t,s}^{DAC} + \sum_{b\in B} wb_{j,b,t}\mathrm{BREM_b} \qquad \forall j\in J, t\in T \qquad \textit{Eq. 38}$$

Similarly, the total amount of CO_2 stored in country *j* in period *t* is given by Eq. 39. This equation considers the CO_2 captured in all the facilities, i.e., the DACCS plants, the biogenic CO_2 captured at the BECCS plants, and the fossil CO_2 captured at the coal and natural gas power plants with

CCS, as well as the CO_2 traded from other countries *j'*. The CO_2 captured at the DACCS facilities (first addend in the equation) accounts for the CO_2 removed from the atmosphere ($rr_{j,t,s}^{DAC}$) and the fossil CO_2 captured during natural gas combustion, estimated from the heating requirements ($\mathrm{HEAT}_\mathrm{s}^\mathrm{DAC}$), the capture efficiency (HCAP) and the direct emissions factor ($\mathrm{DAC}^\mathrm{HEMISSIONS}$). The CO_2 stored from power plants (BECCS, coal CCS and natural gas CCS) is estimated from the CO_2 captured post-combustion, using parameters $\mathrm{STO}_\mathrm{b}^\mathrm{B}$ and $\mathrm{STO}_\mathrm{j,i}^\mathrm{Elec}$. Finally, the CO_2 captured in other countries *j'* and traded to country *j* to be geologically stored is provided by variable $r_{j,j',t}^{Sto}$.

$$\sum_{j'\in GS_{j'}} r_{j,j',t}^{Sto} = \sum_{s\in S} rr_{j,t,s}^{DAC}\left(1 + \mathrm{HEAT}_\mathrm{s}^\mathrm{DAC}\cdot \mathrm{HCAP}\cdot \mathrm{DAC}^\mathrm{HEMISSIONS}\right) + \\ + \sum_{b\in B} bb_{j,b,t}^{BECCS}\mathrm{STO}_\mathrm{b}^\mathrm{B} + \\ + \sum_{i=Coal\ CCS \vee NG\ CCS} gen_{j,i,t}^{Tech}\mathrm{STO}_\mathrm{j,i}^\mathrm{Elec} \qquad \forall j\in J, t\in T \qquad \text{Eq. 39}$$

Eq. 40 ensures that the total amount of captured CO_2 sent to the geological sites in country *j* cannot exceed the geological capacity in each country *j* ($\mathrm{STO}_\mathrm{j}^\mathrm{Cap}$).

$$\sum_{j'\in J}\sum_{t\in T} r_{j',j,t}^{Sto} \le \mathrm{STO}_\mathrm{j}^\mathrm{Cap} \qquad \forall j\in GS_j \qquad \text{Eq. 40}$$

The installed capacity of DACCS ($dac_{j,s,t}^{Avail}$) is given by the capacity expansions taking place in previous periods, as shown in Eq. 41 ($dac_{j,s,t'}^{Exp}$). This available capacity limits the annual amount of CO_2 removed from the atmosphere ($rr_{j,t,s}^{DAC}$ in Gt/yr), as shown in Eq. 42.

$$dac_{j,s,t}^{Avail} = \sum_{t'=t-DAC^{Life}+1}^{t'=t} dac_{j,s,t'}^{Exp} \qquad \forall j\in J, s\in S, t\in T \qquad \text{Eq. 41}$$

$$rr_{j,t,s}^{DAC} \le dac_{j,s,t}^{Avail}\mathrm{DPER} \qquad \forall j\in J, s\in S, t\in T \qquad \text{Eq. 42}$$

1.2.3. Cost equations

Similarly, as with the emissions, Eq. 43 determines the total costs in each country *j* and period *t* from the costs of power generation, excluding the biomass-based technologies ($c_{j,t}^{Power}$), plus the DACCS cost ($c_{j,t}^{DACCS}$), the costs of the biomass-based technologies (Biomass w/o CCS and BECCS) ($c_{j,t}^{BECCS}$), and the expenditures linked to natural gas transportation ($c_{j,t}^{ht}$) and CO_2 transportation and injection in geological sites ($c_{j,t}^{CO2t}$).

$$c_{j,t}^{Country} = c_{j,t}^{Power} + c_{j,t}^{DACCS} + c_{j,t}^{BT} + c_{j,t}^{ht} + c_{j,t}^{CO2t} \qquad \forall j\in J, t\in T \qquad \text{Eq. 43}$$

Eq. 44 computes the costs of the power technologies in each county *j* and period *t* (excluding the biomass-based technologies). The capital expenditures consider the expected capital investment during the horizon ($\mathrm{CAPEX}_\mathrm{i,t}$), which is annualized using the capital recovery factor ($\mathrm{CRF}_\mathrm{j,i}$)estimated considering uniform weighted average costs of capital (WACC) during the lifetime of the technology (UL_i). The WACC represents the discount rate in the net present value calculations (Eq. 55 in section 2.2.3 Cost parameters). The operational costs include the fix costs

($\mathrm{OPEX}_{\mathrm{i,t}}^{\mathrm{Fix}}$) linked to the capacity installed $cap_{j,i,t}^{Avail}$ (e.g., refurbishment costs) and the variable costs ($\mathrm{OPEX}_{\mathrm{i,t}}^{\mathrm{Var}}$). The latter are production-related costs (excluding fuel costs) that depend on the power generated ($gen_{j,i,t}^{Tech}$). Finally, the fuel costs ($\mathrm{FC}_{\mathrm{i}}^{\mathrm{Fuel}}$) are linked to electricity generation ($gen_{j,i,t}^{Tech}$). Note that this term is zero for renewable power technologies (e.g., zero fuel costs for wind or solar).

$$c_{j,t}^{Power} = \sum_{i \notin BT_i} \Big(\mathrm{CAPEX_{i,t}CRF_{j,i}} cap_{j,i,t}^{Exp} \mathrm{UL_i DPER} + \mathrm{OPEX}_{\mathrm{i,t}}^{\mathrm{Fix}} cap_{j,i,t}^{Avail} + \mathrm{OPEX}_{\mathrm{i,t}}^{\mathrm{Var}} gen_{j,i,t}^{Tech} \Big) + \sum_{i \notin BT_i} gen_{j,i,t}^{Tech} \mathrm{FC}_{\mathrm{i}}^{\mathrm{Fuel}} \qquad \forall j \in J, t \in T \qquad \textit{Eq. 44}$$

The costs associated with the DACCS facilities (Eq. 45) include the capital expenditures, non-energy operational and maintenance costs, and the cost related to the heating requirements from natural gas. The capital expenditures for every technology *s* and period *t* are based on projections ($\mathrm{CAPEX}_{\mathrm{s,t}}^{\mathrm{DAC}}$) that are annualized considering a constant capital recovery factor ($\mathrm{CRF}^{\mathrm{DAC}}$) and the expected lifetime of the DACCS technologies ($\mathrm{DAC}^{\mathrm{Life}}$). The non-energy operational expenditures ($\mathrm{OPEX}_{\mathrm{s,t}}^{\mathrm{DAC}}$) include fix and variable costs (e.g., water, labor, and make-up chemicals), linked to the amount of CO_2 removed ($rr_{j,t,s}^{DAC}$). The variable costs related to the natural gas consumption are calculated from the heating needs ($\mathrm{HEAT}_{\mathrm{s}}^{\mathrm{DAC}}$) per mass of CO_2 removed ($rr_{j,t,s}^{DAC}$) and the associated unitary cost ($\mathrm{COST}_{\mathrm{j}}^{\mathrm{Heat}}$).

$$c_{j,t}^{DACCS} = \sum_{s \in S} \Big(\mathrm{CAPEX}_{\mathrm{s,t}}^{\mathrm{DAC}} dac_{j,s,t}^{Exp} \mathrm{CRF^{DAC} DAC^{Life} DPER} + \mathrm{OPEX}_{\mathrm{s,t}}^{\mathrm{DAC}} rr_{j,t,s}^{DAC} \Big) + \sum_{s \in S} rr_{j,t,s}^{DAC} \mathrm{HEAT}_{\mathrm{s}}^{\mathrm{DAC}} \mathrm{COST}_{\mathrm{j}}^{\mathrm{Heat}} \qquad \forall j \in J, t \in T \qquad \textit{Eq. 45}$$

The costs for the bio-based technologies (Biomass and BECCS included in the set *BT*) are provided in Eq. 46, which accounts for the capital and operational expenditures, the biomass raw material costs, and the costs associated with the transportation of pellets within and between countries. The capital and operational expenditure are calculated as in Eq. 41, similarly as done for the other power technologies. Here the costs of each biomass feedstock are determined from the pellets of each type *b* burnt at both Biomass w/o CCS and BECCS plants ($bb_{j,b,t}^{Total}$), and the unitary costs of biomass feedstock linked to the type of biomass *b* combusted ($\mathrm{FC}_{\mathrm{b}}^{\mathrm{Bio}}$). Finally, the costs of biomass transportation in country *j* and period *t* consider the imports from other countries *j'* and the within-country transportation from the field to the power plant. The former costs are computed from the amount of biomass traded from country *j'* to country *j* ($tb_{j',j,b,t}$), the distance between countries ($\mathrm{DC}_{\mathrm{j',j}}$) and the unitary cost of the transportation ($\mathrm{COST}^{\mathrm{BTrans}}$). The latter term considers the biomass pellets produced and consumed within the country *j* (e.g., biomass produced minus exports), the internal distance from the pelleting plant to the power plant ($\mathrm{DC}_{\mathrm{j,j}}$) and the unitary transportation cost ($\mathrm{COST}^{\mathrm{BTrans}}$).

$$c_{j,t}^{BT} = \sum_{i \in BT_i} \Big(\mathrm{CAPEX_{i,t}CRF_{j,i}} cap_{j,i,t}^{Exp} \mathrm{UL_i DPER} + \mathrm{OPEX}_{\mathrm{i,t}}^{\mathrm{Fix}} cap_{j,i,t}^{Avail} + \mathrm{OPEX}_{\mathrm{i,t}}^{\mathrm{Var}} gen_{j,i,t}^{Tech} \Big) + \sum_{b \in B} bb_{j,b,t}^{Total} \mathrm{FC}_{\mathrm{b}}^{\mathrm{Bio}} \qquad \textit{Eq. 46}$$

$$+\sum_{j' \in J}\sum_{b \in B} tb_{j',j,b,t}\mathrm{DC_{j',j}}\mathrm{COST^{BTrans}}$$

$$+\sum_{b \in B}\left(db_{j,b,t} - \sum_{j' \in J} tb_{j,j',b,t}\right)\mathrm{DC_{j,j}}\mathrm{COST^{BTrans}}$$

$$\forall j \in J, t \in T$$

Eq. 47 provides the costs associated with the natural gas transportation from Russia to the EU to cover the heating needs of DACCS and natural gas power plants (w/ and w/o CCS). Note that the transportation costs of natural gas between EU countries are omitted because they are included in the fuel costs of natural gas in Eq. 44. These costs are calculated considering the amount of natural gas traded from Russia to the EU countries ($import_{j,t}^{Russia}$), the higher heating value of natural gas ($\mathrm{HHV^{NG}}$), the distance between countries ($\mathrm{DI_j}$) and the unitary transportation cost via pipeline ($\mathrm{COST^{NGTrans}}$).

$$c_{j,t}^{ht} = \frac{import_{j,t}^{Russia}}{\mathrm{HHV^{NG}}}\mathrm{COST^{Pipetrans}}\mathrm{DI_j} \qquad \forall j \in J, t \in T \qquad \textit{Eq. 47}$$

Finally, Eq. 48 provides the costs in each country *j* and period *t* associated with the transportation and injection of the captured CO_2. The transportation costs consider the total CO_2 captured from BECCS, DACCS and power plants with CCS (variable $r_{j,j',t}^{Sto}$), the distance from the capture plants to the geological sites ($\mathrm{DGS_{j,j'}}$) and the unitary costs of transporting CO_2 via pipelines ($\mathrm{COST^{NGTrans}}$). The costs related to the CO_2 injection into wells consider the amount of CO_2 to be stored ($r_{j,j',t}^{Sto}$) and the unitary injection cost ($\mathrm{COST^{Injec}}$).

$$c_{j,t}^{CO2t} = \sum_{j' \in GS_{j'}}\left(r_{j,j',t}^{Sto}\mathrm{COST^{Pipetrans}}\mathrm{DGS_{j,j'}} + r_{j,j',t}^{Sto}\mathrm{COST^{Injec}}\right) \ \forall j \in J, t \in T \qquad \textit{Eq. 48}$$

1.2.4. Modeling of inactive periods

RAPID allows us to explore the consequences of delaying the deployment of BECCS and DACCS. Hence, Eq. 49 and Eq. 50, respectively, ensure that during inactive periods –selected by the modeler with the set *PI*– BECCS and DACCS cannot be deployed.

$$cap_{j,i,t}^{Avail} = 0 \qquad \forall j \in J, i = BECCS, t \in PI_t \qquad \textit{Eq. 49}$$

$$dac_{j,s,t}^{Avail} = 0 \qquad \forall j \in J, s \in S, t \in PI_t \qquad \textit{Eq. 50}$$

1.2.5. Objective functions

RAPID maximizes the net negative emissions balance (M1) or minimizes the system's costs to meet a given target on net CDR (M2).

The environmental objective function –to be minimized– accounts for the net balance of CO_2 emissions in the system. In essence, the CO_2 emissions balance subtracts, from the positive life cycle emissions in all countries *j* and periods *t* ($e_{j,t}^{Country}$), the CO_2 emissions removed from the atmosphere, modeled as a negative entry in the system ($r_{j,t}^{Total}$, as determined in Eq. 38).

$$(M1) \qquad min\ obj^{Env} = \sum_{j \in J} \sum_{t \in T} e_{j,t}^{Country} - \sum_{j \in J} \sum_{t \in T} r_{j,t}^{Total} \qquad \textit{Eq. 51}$$

$$s.t. \quad constraints\ Eqs. 1 - 50$$

The economic objective function (Eq. 52) quantifies the total costs of the system from the cost in countries *j* in all periods *t* in 2020-2100 ($c_{j,t}^{Country}$). We also add half of the CAPEX of the plants installed at the beginning of the horizon, assuming their age at that time already matches the midpoint of their useful life (second addend in the equation). Note that the OPEX expenditures of the plants already installed are also accounted for through the first term, as defined in Eqs. 43-48. Moreover, Eq. 53 imposes a target (α) on the net CO_2 balance to be provided by the system, which can be either positive, negative (to deliver an amount of CDR), or zero (CO_2-neutrality).

$$(M2) \qquad min\ obj^{Eco} = \sum_{j \in J} \sum_{t \in T} c_{j,t}^{Country} + \sum_{i \in I} \sum_{j \in J} \left(\frac{CAP_{j,i}^{Today}}{2} \mathrm{CAPEX}_{\mathrm{i,p_1}} \mathrm{crf}_{\mathrm{j,i}} \mathrm{UL}_{\mathrm{i}} \mathrm{DPER} \right) \qquad \textit{Eq. 52}$$

$$s.t. \quad obj^{Env} \le \alpha \qquad \textit{Eq. 53}$$

$$constraints\ Eqs. 1 - 51$$

2. Supplementary data

This section provides the values of all the parameters and describes some of the modeling assumptions.

2.1. Sets

The elements of each main set are shown in **Supplementary Table 3**.

Supplementary Table 3. Elements of the main sets.

Set	Elements
T	p_1, ..., p_{16}.
J	Countries of the EU-28.
I	Wind onshore, Wind offshore, Hydro run-of-river, Hydro reservoir, Geothermal, Solar photovoltaic open ground, Solar photovoltaic roof, Solar thermal parabolic, Coal, Coal + CCS, Natural Gas, Natural Gas + CCS, Nuclear, Biomass, BECCS.
B	Miscanthus, Miscanthus + CCS, Switchgrass, Switchgrass + CCS, Willow, Willow + CCS, Straw Residues, Straw Residues + CCS, Agricultural prunings, Agricultural prunings + CCS, Forest residues, Forest residues + CCS.
S	Type A (only heat), Type C (electricity and heat).

The subsets defined from these sets are shown in **Supplementary Table 4**.

Supplementary Table 4. Elements of the subsets.

Set	Elements
PI_t	Number of inactive periods. PI can be an empty set or comprise any number of elements between p_1 and p_{16} in consecutive order, starting with p_1.
GS_j	Countries of the EU-28 with CO_2 geological storage capacity.
TD_i	All technologies excluding Wind Onshore, Wind Offshore, Solar photovoltaic open ground, Solar photovoltaic roof.
RE_i	Wind onshore, Wind offshore, Hydro run-of-river, Hydro reservoir, Geothermal, Solar photovoltaic open ground, Solar photovoltaic roof, Solar thermal parabolic.
BC_b	Miscanthus, Switchgrass, Willow.
BR_b	Straw residues, Agricultural prunings, Forest residues.
BT_i	Biomass, BECCS.

2.2. Data description and assumptions

2.2.1. Distance Parameters

Distances are computed based on the centroids of the countries, considering their latitude and longitude. These data, extracted from developers.google[3], are used to define the values of parameters $\mathrm{DC}_{j,j'}$, DI_j and $\mathrm{DGS}_{j,j'}$. A 100 km distance within each country is assumed for domestic consumption of biomass resources and domestic storage of CO_2 emissions (i.e., biomass transportation from the field to the power plant, parameter $\mathrm{DC}_{j,j}$, and CO_2 transported from the capture plant to the geological site, parameter $\mathrm{DGS}_{j,j}$).

2.2.2. DAC Parameters

For the DACCS technology, the following parameters are used (**Supplementary Table 5**).

Supplementary Table 5. DACCS parameters.

Parameter	Value	Source
$\mathrm{HEAT}_s^{\mathrm{DAC}}$	Type A: 8.81 GJ/tCO_2<br>Type C: 5.25 GJ/tCO_2	Keith et al.[4]
$\mathrm{ELEC}_s^{\mathrm{DAC}}$	Type A: 0 kWh/tCO_2<br>Type C: 366 kWh/tCO_2	Keith et al.[4]
HCAP	90 %	Keith et al.[4]
$\mathrm{DAC}^{\mathrm{HEMISSIONS}}$	4.98x10^{-3} kgCO_2/MJ	#Estimated
$\mathrm{DAC}^{\mathrm{NGEXTRACT}}$	2.46x10^{-3} kgCO_2/MJ	Wernet et al.[5]
$\mathrm{DACLIFE}$	30 yr	Child et al.[6]
$\mathrm{INITIAL}^{\mathrm{DAC}}$	1 MtCO_2/yr	

*Type A refers to the DACCS technology with only heating requirements, while Type C refers to the DACCS technology with both heating and power requirements. Both types use an aqueous KOH sorbent.

#The CO_2 emissions released during the combustion of natural gas for heating are estimated in Eq. 54.

The initial capacity of DACCS is set to 1 Mton/yr, reflecting the current ambition of the Carbon Engineering plant in Texas, still under construction.

Eq. 54 provides the CO_2 emissions linked to the combustion of natural gas to power DACCS (parameter $\mathrm{DAC}^{\mathrm{HEMISSIONS}}$) from the stoichiometric relationship between CH_4 and CO_2. Here, $\mathrm{MW}^{\mathrm{CH_4}}$ and $\mathrm{MW}^{\mathrm{CO_2}}$ refer to the molecular weights of CH_4 and CO_2, respectively, and $\mathrm{HHV}^{\mathrm{NG}}$ corresponds to the higher heating value of natural gas, i.e., 55.25 MJ/kg.

$$\mathrm{DAC}^{\mathrm{HEMISSIONS}} = \frac{\mathrm{MW}^{\mathrm{CO_2}}}{\mathrm{HHV}^{\mathrm{NG}} \cdot \mathrm{MW}^{\mathrm{CH_4}}} \quad \textit{Eq. 54}$$

2.2.3. Cost parameters

The CAPEX values of the power technologies ($\mathrm{CAPEX}_{i,t}$) are shown in **Supplementary Table 6**. **Supplementary Table 7** displays the variable operating costs ($\mathrm{OPEX}_{i,t}^{\mathrm{Var}}$) –excluding the costs associated with fuel consumption, provided in **Supplementary Table 8** for the technologies, and in **Supplementary Table 9** for the biomass–. Besides the CAPEX data in **Supplementary Table 6**, our sensitivity analysis considers the lower and upper bounds[7] in **Supplementary Table 10** and **Supplementary Table 11**, respectively. We consider learning rates for the CAPEX costs as estimated in Carlsson et al.[7] based on the technologies' installed capacity; these learning rates affect the OPEX as well, since they are calculated as a percentage of the CAPEX. To estimate the Levelized cost of electricity, we assume a fuel consumption rate per kWh of 0.44 kg coal, 0.19 m^3 of natural gas, and $2.46 \cdot 10^{-6}$ kg of uranium –taken from the Ecoinvent 3.5 database–[5]. The coal and natural gas consumption rates for the CCS scenarios assume an increase in fuel consumption (relative to the non-CCS case) of 31.2% and 16.3%, respectively, based on ref[8]. We assume a price of 60 2019\$/ton for coal[9], 7.60 2019€/GJ for natural gas (HHV)[10], and 73.74 2018€/kg for uranium[11]. Moreover, the biomass costs are sourced from de Wit et al.[12]. Further details on the biomass sources are given in sections 2.2.4 and 2.2.5, and in **Supplementary Table 25**.

The $\mathrm{OPEX}^{\mathrm{FIX}}$ parameter is calculated from the fixed operating costs without refurbishment (**Supplementary Table 12**), and the refurbishment fixed operating costs taken from the original reference, spread over the useful life of the corresponding technology (**Supplementary Table 13**).

The costs data for the power technologies are taken from Carlsson et al.[7], except for the BECCS costs which were obtained from Cabezzali et al.;[13]. These data are assumed to remain constant over time. The cost parameters for those periods missing in the tables are assumed to have the same values as those reported.

All cost data are updated to 2015, considering a 2% inflation rate. The exchange rate from dollars to euros is 1.09 \$/€.

Supplementary Table 6. Capital expenditures ($\mathrm{CAPEX}_{i,t}$) [2013€/KW][7].

Technology	2020 (p_1)	2030 (p_3)	2040 (p_5)	2050 (p_6)
Wind onshore	1,350	1,300	1,200	1,100
Wind offshore	2,880	2,580	2,380	2,280
Hydro run-of-river	5,600	5,620	5,620	5,620
Hydro reservoir	3,360	3,370	3,370	3,370
Geothermal	4,970	4,470	4,020	3,610
Solar photovoltaic open ground	800	640	580	520
Solar photovoltaic roof	1100	990	930	880
Solar parabolic thermal	4,500	3,800	3,500	3,400
Coal	1,600	1,600	1,600	1,600
Natural Gas	850	850	850	850
Nuclear	6,300	5,750	5,350	5,300
Coal + CCS	2,700	2,550	2,550	2,550

Natural Gas + CCS	1,500	1,500	1,500	1,500
Biomass	2,620	2,330	2,060	1,830
BECCS	3,331	3,331	3,331	3,331

Supplementary Table 7.Operational expenditures ($OPEX^{VAR}$) [2013€/KWh] [7].

Technology	$OPEX^{VAR}$
Wind onshore	0
Wind offshore	0
Hydro run-of-river	$5.00x10^{-3}$
Hydro reservoir	$5.00x10^{-3}$
Geothermal	0
Solar photovoltaic open ground	0
Solar photovoltaic roof	0
Solar parabolic thermal	$8.00x10^{-3}$
Coal	$3.60x10^{-3}$
Natural Gas	$2.00x10^{-3}$
Nuclear	$2.50x10^{-3}$
Coal + CCS	$5.50x10^{-3}$
Natural Gas + CCS	$4.00x10^{-3}$
Biomass	$3.80x10^{-3}$
BECCS	$9.95x10^{-3}$

Supplementary *Table* 8. Fuel contribution to the electricity cost (FC_i^{fuel}) [2015€/kWh].

Fuel	w/o CCS	with CCS
Coal	$2.17x10^{-2}$	$2.85x10^{-2}$
Natural gas	$4.95x10^{-2}$	$5.81x10^{-2}$
Uranium	$1.71x10^{-4}$	-

**The fuel contribution is calculated considering the Ecoinvent activities "Electricity, high voltage {RoW}| electricity production, hard coal | Cut-off, U", "Electricity, high voltage {RoW}| electricity production, natural gas, combined cycle power plant | Cut-off, U" and "Nuclear fuel element, for pressure water reactor, UO2 4.2% & MOX {GLO}| market for | Cut-off, U" as well as the fuel price and the increased fuel requirement for the case of the CCS technologies*

Supplementary Table 9. Fuel contribution to the biomass raw materials (FC_b^{Bio}) [2010€/kg (db)][12].

Fuel	FC_b^{bio}
Miscanthus	$7.14\ x10^{-2}$
Switchgrass	$5.93\ x10^{-2}$
Willow	$5.99\ x10^{-2}$
Straw residues	$6.40\ x10^{-2}$
Agricultural prunings	$5.46\ x10^{-2}$
Forest residues	$5.46\ x10^{-2}$

Supplementary Table 10. Low CAPEX [2013€/kW].

Technology	2020	2030	2040	2050
Wind onshore	1,100	1,000	900	800
Wind offshore	2,580	2,280	2,080	1,790

Hydro run-of-river	2,540	2,560	2,560	2,560
Hydro reservoir	1,220	1,230	1,230	1,230
Geothermal	250	2,500	2,500	2,500
Solar photovoltaic open ground	650	520	470	420
Solar photovoltaic roof	950	850	810	760
Solar parabolic thermal	3,300	3,000	2,800	2,600
Coal	1,550	1,550	1,550	1,550
Natural Gas	700	700	700	700
Nuclear	3,850	3,650	3,400	3,350
Coal + CCS	2,340	2,210	2,210	2,210
Natural Gas + CCS	1,250	1,250	1,250	1,250
Biomass	1,540	1,350	1,190	1,040

***Supplementary Table* 11**. High CAPEX [2013€/kW].

Technology	2020	2030	2040	2050
Wind onshore	2,000	1,800	1,700	1,700
Wind offshore	4,270	3,970	3,470	3,270
Hydro run-of-river	8,150	8,180	8,180	8,180
Hydro reservoir	4,580	4,600	4,600	4,600
Geothermal	5,370	4,870	4,420	4,010
Solar photovoltaic open ground	900	720	650	580
Solar photovoltaic roof	1250	1120	1060	1000
Solar parabolic thermal	6,000	5,000	4,500	4,000
Coal	1,700	1,700	1,700	1,700
Natural Gas	950	950	950	950
Nuclear	7,750	7,100	6,550	6,500
Coal + CCS	3,020	2,850	2,850	2,850
Natural Gas + CCS	1,750	1,750	1,750	1,750
Biomass	3,170	2,780	2,440	2,140

Supplementary Table 12. Fixed operating costs, excluding refurbishment ($\mathrm{OPEX}_{i,t}^{\mathrm{Fix}}$) [2013€/kW/yr] [7,13].

Technology	2020	2030	2040	2050
Wind onshore	32.40	28.60	22.80	18.70
Wind offshore	92.16	77.40	66.64	52.44
Hydro run-of-river	84.00	84.30	84.30	84.30
Hydro reservoir	50.40	50.55	50.55	50.55
Geothermal	79.52	80.46	80.40	79.42
Solar photovoltaic open ground	13.60	10.88	9.86	8.84
Solar photovoltaic roof	22.00	19.80	18.60	17.60
Solar parabolic thermal	180.00	152.00	140.00	136.00
Coal	40.00	40.00	40.00	40.00
Natural Gas	21.25	21.25	21.25	21.25
Nuclear	126.00	115.00	107.00	106.00
Coal + CCS	67.50	63.75	63.75	63.75
Natural Gas + CCS	37.50	37.50	37.50	37.50

Biomass	47.16	41.94	37.08	32.94
BECCS	109.92	109.92	109.92	109.92

Supplementary Table 13. Refurbishment fixed operating costs [2013€/kW/yr].

Technology	2020 (p_1)	2030 (p_3)	2040 (p_5)	2050 (p_6)
Hydro run-of-river	168.00	168.60	168.60	168.60
Hydro reservoir	100.80	101.10	101.10	101.10
Nuclear	0	0	0	106.00
Biomass	23.58	20.97	18.54	16.47

For the DACCS cost, we use data from Keith et al.[4] and apply the learning curve from Child et al.[6], as shown in **Supplementary Table 14**. Note that these costs omit the cost for transporting the CO_2 via pipeline and the cost of injection into geological sites, shown in **Supplementary Table 18**.

Supplementary Table 14. Cost parameters for DACCS, $\mathrm{CAPEX}_{s,t}^{\mathrm{DAC}}$ [2015$/(t/yr)] and $\mathrm{OPEX}_{s,t}^{\mathrm{DAC}}$ [2015$/t] [4,6].

Parameter	2020	2025	2030	2035	2040	2045	2050
$CAPEX^{DAC}$ (s=A)	1,146	1,016	886	757	627	497	368
$CAPEX^{DAC}$ (s=C)	694	615	537	458	380	301	223
$OPEX^{DAC}$(s=A)	30	30	30	30	30	30	30
$OPEX^{DAC}$(s=C)	26	26	26	26	26	26	26

*The tons refer to CO_2 removed from the atmosphere.

The cost of heating was sourced from Eurostat[10], and the data per country is shown in **Supplementary Table 15**.

Supplementary Table 15.Cost of the heating from natural gas ($COST^{HEAT}$) [2019€/kWh].

Country	$COST^{HEAT}$
Austria	2.64×10^{-2}
Belgium	2.19×10^{-2}
Bulgaria	2.97×10^{-2}
Cyprus	2.79×10^{-2}
Czechia	2.80×10^{-2}
Germany	2.75×10^{-2}
Denmark	2.43×10^{-2}
Spain	2.93×10^{-2}
Estonia	2.88×10^{-2}
Finland	4.69×10^{-2}
France	3.07×10^{-2}
United Kingdom	2.65×10^{-2}
Greece	2.72×10^{-2}
Hungary	2.70×10^{-2}
Ireland	3.11×10^{-2}
Italy	2.77×10^{-2}
Lithuania	2.81×10^{-2}

Luxembourg	3.30×10^{-2}
Latvia	3.04×10^{-2}
Malta	2.79×10^{-2}
Netherlands	2.23×10^{-2}
Poland	3.37×10^{-2}
Portugal	3.17×10^{-2}
Romania	3.10×10^{-2}
Croatia	2.93×10^{-2}
Slovakia	3.29×10^{-2}
Slovenia	2.84×10^{-2}
Sweden	3.15×10^{-2}

The capital recovery factor parameter (CRF) can be obtained from Eq. 55:

$$\mathrm{CRF} = \frac{\mathrm{WACC} \cdot (1 + \mathrm{WACC})^{\mathrm{N_t}}}{(1 + \mathrm{WACC})^{\mathrm{N_t}} - 1} \qquad \textit{Eq. 55}$$

Where WACC refers to the weighted average cost of capital and $\mathrm{N_t}$ to the useful life in years. We consider a WACC of 7% and the lifetime of each technology evaluated. When available, region-specific data of the CRF was employed[6,14] as shown in **Supplementary Table 16;** otherwise, values estimated with Eq. 55 were employed instead (**Supplementary Table 17**).

Supplementary Table 16. Regionalized capital recovery factor ($\mathrm{CRF_{i,j}}$).

Country	Wind onshore	Wind offshore	Solar Photovoltaic and Thermal Parabolic
Austria	7.75×10^{-2}	9.61×10^{-2}	6.80×10^{-2}
Belgium	5.94×10^{-2}	7.45×10^{-2}	4.78×10^{-2}
Bulgaria	1.05×10^{-1}	9.61×10^{-2}	6.80×10^{-2}
Cyprus	1.05×10^{-1}	9.61×10^{-2}	6.80×10^{-2}
Czechia	8.89×10^{-2}	9.61×10^{-2}	6.80×10^{-2}
Germany	5.74×10^{-2}	7.90×10^{-2}	4.91×10^{-2}
Denmark	7.17×10^{-2}	9.21×10^{-2}	6.80×10^{-2}
Spain	1.05×10^{-1}	9.61×10^{-2}	6.80×10^{-2}
Estonia	1.03×10^{-1}	9.61×10^{-2}	6.80×10^{-2}
Finland	7.75×10^{-2}	9.61×10^{-2}	6.80×10^{-2}
France	7.17×10^{-2}	9.61×10^{-2}	6.80×10^{-2}
United Kingdom	8.01×10^{-2}	1.33×10^{-1}	6.80×10^{-2}
Greece	1.25×10^{-1}	9.61×10^{-2}	1.22×10^{-1}
Hungary	1.16×10^{-1}	9.61×10^{-2}	6.80×10^{-2}
Ireland	9.69×10^{-2}	9.61×10^{-2}	6.80×10^{-2}
Italy	8.89×10^{-2}	9.61×10^{-2}	6.80×10^{-2}
Lithuania	9.29×10^{-2}	9.61×10^{-2}	6.80×10^{-2}
Luxembourg	8.81×10^{-2}	9.61×10^{-2}	6.80×10^{-2}
Latvia	9.93×10^{-2}	9.61×10^{-2}	6.80×10^{-2}
Malta	8.81×10^{-2}	9.61×10^{-2}	6.80×10^{-2}
Netherlands	7.60×10^{-2}	1.09×10^{-1}	6.80×10^{-2}
Poland	9.93×10^{-2}	9.61×10^{-2}	6.80×10^{-2}
Portugal	8.89×10^{-2}	9.61×10^{-2}	6.80×10^{-2}
Romania	1.14×10^{-1}	9.61×10^{-2}	6.80×10^{-2}
Croatia	1.22×10^{-1}	9.61×10^{-2}	6.80×10^{-2}

Slovakia	$8.97x10^{-2}$	$9.61x10^{-2}$	$6.80x10^{-2}$
Slovenia	$1.14x10^{-1}$	$9.61x10^{-2}$	$6.80x10^{-2}$
Sweden	$9.05x10^{-2}$	$9.61x10^{-2}$	$6.80x10^{-2}$

Supplementary Table 17. CRF considering an average 7% WACC[6].

Technology	Value
Hydro	$7.12x10^{-2}$
Geothermal	$7.50x10^{-2}$
Coal	$7.50x10^{-2}$
Natural Gas	$7.72x10^{-2}$
Nuclear	$7.50x10^{-2}$
Biomass	$8.06x10^{-2}$
BECCS	$8.06x10^{-2}$
DAC	$8.06x10^{-2}$

The remaining cost parameters are shown in **Supplementary Table 18**, including the cost for natural gas transportation, biomass transport, injection, and the inflation rate.

Supplementary Table 18. Other cost parameters.

Parameter	Value
$COST^{PIPETRANS}$	5.1710^{-2} 2010€/tkm*[15]
$COST^{BTRANS}$	$2.30x10^{-2}$ 2015€/tkm*[16,17]
$COST^{INJEC}$	20.00 2015\$/tCO_2[18]
IF	2 %

*tkm is the abbreviation of ton-kilometer, i.e., transport of one ton of goods over one kilometer with a particular transportation media.

We consider a constant inflation rate (IF) of 2% per year. This value is around the median value for Europe between 2010 and 2020 [19]. All costs in the manuscript are given in €2015. Whenever necessary, a conversion factor of 1.09 $/€ from 2015US$ to 2015€ was applied.

2.2.4. Emission parameters

The life cycle CO_2 emissions for the power technologies and biomass and CO_2 supply chain activities are taken from the Ecoinvent v3.5 database[5]. All emissions data were sourced considering the “Allocation at the point of substitution” (APOS) system model. The Ecoinvent database v3.5[5] distinguishes between biogenic and fossil CO_2 flows. The biogenic carbon uptake and the biogenic carbon releases are often unbalanced at the level of activity due to the allocation methods implemented.

Our reference system relies on biomass resources as the primary feedstock for the BECCS and biomass power plants. Hence, we need to adjust the carbon balance so as to provide credits to the CO_2 removed from the atmosphere, ensuring its long-term storage. Similarly, our work also considers the direct removal of CO_2 from the atmosphere taking place in the DACCS plants.

Accordingly, the biogenic carbon and the CO_2 captured with DACCS were tracked manually to carry out a tailored CO_2 balance. Hence, we first excluded all the biogenic carbon from the inventory data in Ecoinvent to consider only the non-biogenic emissions to air. This is a common assumption in most LCIA methods, as the CO_2 uptake by biomass via photosynthesis will be eventually released back into the air. The CO_2 uptake from the atmosphere via photosynthesis

or chemical reactions is modeled as a negative flow of CO_2 entering the system. For the biomass resources (i.e., energy crops and residues from agriculture and forestry activities), the CO_2 uptake is estimated from the carbon and water content (see **Supplementary Table 28**). These CO_2 flows are tracked along the supply chains by accounting for the flows leaving the system as positive flows (e.g., biomass losses, uncaptured CO_2, or other leakages).

Therefore, we consider only the non-biogenic emissions to air labeled in Ecoinvent v3.5[5] as follows:

- Carbon dioxide, from soil or biomass stock, non-urban air or from high stacks.
- Carbon dioxide, fossil, non-urban air or from high stacks.
- Carbon dioxide, fossil, unspecified.
- Carbon dioxide, fossil, urban air close to ground.
- Carbon dioxide, fossil, lower stratosphere + upper troposphere.
- Carbon dioxide, from soil or biomass stock, unspecified.

The names of the activities used in Ecoinvent v3.5[5] are as follows:

- Wind onshore: electricity, high voltage, electricity production, wind, 1-3MW turbine, onshore.
- Wind offshore: electricity, high voltage, electricity production, wind, 1-3MW turbine, offshore.
- Hydro run-of-river: electricity, high voltage, electricity production, hydro, run-of-river.
- Hydro reservoir: electricity, high voltage, electricity production, hydro, reservoir, non-alpine region.
- Geothermal: electricity, high voltage, electricity production, deep geothermal.
- Solar photovoltaic open ground: electricity production, photovoltaic, 570kWp open ground installation, multi-Si.
- Solar photovoltaic roof: electricity production, photovoltaic, 3kWp flat-roof installation, multi-Si.
- Solar thermal parabolic: electricity, high voltage, electricity production, solar thermal parabolic trough, 50 MW.
- Coal: electricity, high voltage, electricity production, hard coal.
- Natural Gas: electricity, high voltage, electricity production, natural gas, combined cycle power plant.
- Nuclear: electricity, high voltage, electricity production, nuclear, pressure water reactor.

The adjusted carbon intensity parameters for the power technologies are shown in **Supplementary Table 19**, where "*" indicates that we considered Rest of the World (RoW) data in the absence of region-specific data.

Supplementary Table 19. Life cycle emissions of the electricity generation technologies ($\mathrm{EM}_{j,i}^{\mathrm{Elec}}$) [$kgCO_2/kWh$].

**Country**	**Wind onshore**	**Wind offshore**	**Hydro run-of-river**	**Hydro reservoir**
Austria	1.53×10^{-2}	1.42×10^{-2}*	3.97×10^{-3}	4.51×10^{-2}*
Belgium	1.37×10^{-2}	1.42×10^{-2}	3.97×10^{-3}	4.51×10^{-2}*
Bulgaria	1.53×10^{-2}	1.42×10^{-2}*	3.97×10^{-3}	4.51×10^{-2}*
Cyprus	2.17×10^{-2}	1.42×10^{-2}*	4.19×10^{-3}*	4.51×10^{-2}*
Czechia	1.71×10^{-2}	1.42×10^{-2}*	3.97×10^{-3}	4.46×10^{-2}

Germany	$1.69x10^{-2}$	$1.42x10^{-2}$	$3.97x10^{-3}$	$4.46x10^{-2}$
Denmark	$1.11x10^{-2}$	$1.42x10^{-2}$	$3.97x10^{-3}$	$4.51x10^{-2}$*
Spain	$1.26x10^{-2}$	$1.42x10^{-2}$	$3.97x10^{-3}$	$4.46x10^{-2}$
Estonia	$1.70x10^{-2}$	$1.42x10^{-2}$	$3.97x10^{-3}$	$4.51x10^{-2}$*
Finland	$1.60x10^{-2}$	$1.42x10^{-2}$	$3.97x10^{-3}$	$4.46x10^{-2}$
France	$1.37x10^{-2}$	$1.42x10^{-2}$	$3.97x10^{-3}$	$4.51x10^{-2}$*
United Kingdom	$1.18x10^{-2}$	$1.42x10^{-2}$	$3.97x10^{-3}$	$4.51x10^{-2}$*
Greece	$1.24x10^{-2}$	$1.42x10^{-2}$*	$3.97x10^{-3}$	$4.51x10^{-2}$*
Hungary	$1.17x10^{-2}$	$1.42x10^{-2}$*	$3.97x10^{-3}$	$4.51x10^{-2}$*
Ireland	$1.19x10^{-2}$	$1.42x10^{-2}$	$3.97x10^{-3}$	$4.51x10^{-2}$*
Italy	$1.66x10^{-2}$	$1.42x10^{-2}$*	$3.97x10^{-3}$	$4.51x10^{-2}$*
Lithuania	$1.14x10^{-2}$	$1.42x10^{-2}$*	$3.97x10^{-3}$	$4.51x10^{-2}$*
Luxembourg	$1.64x10^{-2}$	$1.42x10^{-2}$*	$3.97x10^{-3}$	$4.51x10^{-2}$*
Latvia	$1.63x10^{-2}$	$1.42x10^{-2}$*	$3.97x10^{-3}$	$4.51x10^{-2}$*
Malta	$1.27x10^{-2}$*	$1.42x10^{-2}$*	$4.19x10^{-3}$*	$4.51x10^{-2}$*
Netherlands	$1.31x10^{-2}$	$1.42x10^{-2}$	$3.97x10^{-3}$	$4.51x10^{-2}$*
Poland	$1.44x10^{-2}$	$1.42x10^{-2}$	$3.97x10^{-3}$	$4.51x10^{-2}$*
Portugal	$1.21x10^{-2}$	$1.42x10^{-2}$	$3.97x10^{-3}$	$4.46x10^{-2}$
Romania	$1.97x10^{-2}$	$1.42x10^{-2}$*	$3.97x10^{-3}$	$4.51x10^{-2}$*
Croatia	$1.50x10^{-2}$	$1.42x10^{-2}$*	$3.97x10^{-3}$	$4.51x10^{-2}$*
Slovakia	$1.37x10^{-2}$	$1.42x10^{-2}$*	$3.97x10^{-3}$	$4.46x10^{-2}$
Slovenia	$1.27x10^{-2}$*	$1.42x10^{-2}$*	$3.97x10^{-3}$	$4.51x10^{-2}$*
Sweden	$1.43x10^{-2}$	$1.42x10^{-2}$*	$3.97x10^{-3}$	$4.46x10^{-2}$

**Country**	**Geothermal**	**Solar photovoltaic open ground**	**Solar photovoltaic roof**	**Solar thermal parabolic**
Austria	$6.87x10^{-2}$	$8.13x10^{-2}$	$8.13x10^{-2}$	$6.14x10^{-2}$*
Belgium	$6.87x10^{-2}$*	$9.32x10^{-2}$	$9.32x10^{-2}$	$6.14x10^{-2}$*
Bulgaria	$6.87x10^{-2}$*	$5.93x10^{-2}$	$5.93x10^{-2}$	$6.14x10^{-2}$*
Cyprus	$6.87x10^{-2}$*	$4.58x10^{-2}$	$4.58x10^{-2}$	$6.14x10^{-2}$*
Czechia	$6.87\ x10^{-2}$	$9.00x10^{-2}$	$9.00x10^{-2}$	$6.14x10^{-2}$*
Germany	$6.87x10^{-2}$	$8.44x10^{-2}$	$8.44x10^{-2}$	$6.14x10^{-2}$*
Denmark	$6.87x10^{-2}$*	$6.76x10^{-2}$	$6.76x10^{-2}$	$6.14x10^{-2}$*
Spain	$6.87x10^{-2}$*	$5.49x10^{-2}$	$5.48x10^{-2}$	$6.19x10^{-2}$
Estonia	$6.87x10^{-2}$*	$6.76x10^{-2}$*	$6.76x10^{-2}$*	$6.14x10^{-2}$*
Finland	$6.87x10^{-2}$*	$6.76x10^{-2}$	$6.76x10^{-2}$	$6.14x10^{-2}$*
France	$6.87x10^{-2}$	$6.96x10^{-2}$	$6.96x10^{-2}$	$6.14x10^{-2}$*
United Kingdom	$6.87x10^{-2}$	$6.76x10^{-2}$	$6.76x10^{-2}$	$6.14x10^{-2}$*
Greece	$6.87x10^{-2}$*	$5.81x10^{-2}$	$5.81x10^{-2}$	$6.14x10^{-2}$*
Hungary	$6.87x10^{-2}$	$6.76x10^{-2}$	$6.76x10^{-2}$	$6.14x10^{-2}$*
Ireland	$6.87x10^{-2}$*	$6.76x10^{-2}$	$6.76x10^{-2}$	$6.14x10^{-2}$*
Italy	$6.87x10^{-2}$	$6.28x10^{-2}$	$6.28x10^{-2}$	$6.14x10^{-2}$*
Lithuania	$6.87x10^{-2}$	$8.28x10^{-2}$	$8.28x10^{-2}$	$6.14x10^{-2}$*
Luxembourg	$6.87x10^{-2}$*	$6.76x10^{-2}$	$6.76x10^{-2}$	$6.14x10^{-2}$*
Latvia	$6.87x10^{-2}$	$6.76x10^{-2}$	$6.76x10^{-2}$	$6.14x10^{-2}$*
Malta	$6.87x10^{-2}$*	$6.76x10^{-2}$	$6.76x10^{-2}$	$6.14x10^{-2}$*
Netherlands	$6.87x10^{-2}$*	$8.28x10^{-2}$	$8.28x10^{-2}$	$6.14x10^{-2}$*
Poland	$6.87x10^{-2}$	$7.57x10^{-2}$	$7.57x10^{-2}$	$6.14x10^{-2}$*
Portugal	$6.87x10^{-2}$	$5.34x10^{-2}$	$5.34x10^{-2}$	$6.14x10^{-2}$*

Romania	$6.87x10^{-2}$*	$6.71x10^{-2}$	$6.70x10^{-2}$	$6.14x10^{-2}$*
Croatia	$6.87x10^{-2}$*	$5.38x10^{-2}$	$5.38x10^{-2}$	$6.14x10^{-2}$*
Slovakia	$6.87x10^{-2}$*	$6.91x10^{-2}$	$6.91x10^{-2}$	$6.14x10^{-2}$*
Slovenia	$6.87x10^{-2}$*	$6.34x10^{-2}$	$6.34x10^{-2}$	$6.14x10^{-2}$*
Sweden	$6.87x10^{-2}$*	$8.48x10^{-2}$	$8.48x10^{-2}$	$6.14x10^{-2}$*

**Country**	**Coal**	**Natural Gas**	**Nuclear**
Austria	$8.87x10^{-1}$	$4.40x10^{-1}$	$1.01x10^{-2}$*
Belgium	1.02	$3.78x10^{-1}$	$1.01x10^{-2}$
Bulgaria	1.77	$3.61x10^{-1}$	$1.01x10^{-2}$
Cyprus	1.00*	$3.47x10^{-1}$	$1.01x10^{-2}$*
Czechia	1.11	$4.23x10^{-1}$	$1.01x10^{-2}$
Germany	$9.78x10^{-1}$	$3.90x10^{-1}$	$9.27x10^{-3}$
Denmark	1.00*	$4.10x10^{-1}$*	$1.01x10^{-2}$*
Spain	1.07	$4.45x10^{-1}$	$1.01x10^{-2}$
Estonia	1.05	$4.10x10^{-1}$*	$1.01x10^{-2}$*
Finland	$9.48x10^{-1}$	$7.26x10^{-1}$	$1.01x10^{-2}$
France	$9.97x10^{-1}$	$5.05x10^{-1}$	$9.53x10^{-3}$
United Kingdom	$9.88x10^{-1}$	$3.43x10^{-1}$	$1.01x10^{-2}$
Greece	1.00*	$5.10x10^{-1}$	$1.01x10^{-2}$*
Hungary	1.00*	$5.04x10^{-1}$	$1.01x10^{-2}$
Ireland	$9.55x10^{-1}$	$3.65x10^{-1}$	$1.01x10^{-2}$*
Italy	$9.99x10^{-1}$	$4.16x10^{-1}$	$1.01x10^{-2}$*
Lithuania	1.00*	$4.10x10^{-1}$*	$1.01x10^{-2}$
Luxembourg	1.00*	$3.61x10^{-1}$	$1.01x10^{-2}$*
Latvia	$8.95x10^{-1}$	$3.61x10^{-1}$	$1.01x10^{-2}$
Malta	1.00*	$3.61x10^{-1}$	$1.01x10^{-2}$*
Netherlands	$9.28x10^{-1}$	$3.54x10^{-1}$	$1.01x10^{-2}$
Poland	1.00*	$3.82x10^{-1}$	$1.01x10^{-2}$*
Portugal	$9.93x10^{-1}$	$4.12x10^{-1}$	$1.01x10^{-2}$*
Romania	1.00*	$3.61x10^{-1}$	$1.24x10^{-2}$
Croatia	1.04	$6.72x10^{-1}$	$1.01x10^{-2}$*
Slovakia	1.00*	$4.68x10^{-1}$	$1.01x10^{-2}$
Slovenia	1.00*	$3.61x10^{-1}$	$1.01x10^{-2}$
Sweden	1.00*	$3.54x10^{-1}$	$1.01x10^{-2}$

*The Rest of the World (RoW) dataset was used due to the activity is not available for the particular location.

To account for the CO_2 captured at fossil fuel power plants with CCS, we considered the direct emissions of fossil plants without CCS reported in Ecoinvent v3.5[5] and presented in **Supplementary Table 20**. The life cycle emissions of coal and natural gas coupled with CCS, shown in **Supplementary Table 21**, are calculated assuming a CO_2 capture rate of 90% relative to the direct emissions without CCS and a surplus of fuel –to power the CCS system– of 31.2% and 16.3% for coal and natural gas plants, respectively[8]

Supplementary Table 20. Direct post-combustion emissions of fossil-based electricity technologies [$kgCO_2/kWh$].

**Country**	**Coal**	**Natural Gas**
Austria	$8.04x10^{-1}$	$3.64x10^{-1}$
Belgium	$9.35x10^{-1}$	$3.35x10^{-1}$
Bulgaria	1.62	$3.24x10^{-1}$

Cyprus	0.96*	3.24×10^{-1}
Czechia	1.00	3.59×10^{-1}
Germany	8.90×10^{-1}	3.41×10^{-1}
Denmark	0.96*	0.38*
Spain	9.28×10^{-1}	3.78×10^{-1}
Estonia	9.58×10^{-1}	0.38*
Finland	8.63×10^{-1}	6.18×10^{-1}
France	9.12×10^{-1}	4.50×10^{-1}
United Kingdom	8.95×10^{-1}	3.23×10^{-1}
Greece	0.96*	4.05×10^{-1}
Hungary	0.96*	4.03×10^{-1}
Ireland	8.65×10^{-1}	3.50×10^{-1}
Italy	9.10×10^{-1}	3.55×10^{-1}
Lithuania	0.96*	0.38*
Luxembourg	0.96*	3.24×10^{-1}
Latvia	8.17×10^{-1}	3.24×10^{-1}
Malta	0.96*	3.24×10^{-1}
Netherlands	8.43×10^{-1}	3.24×10^{-1}
Poland	0.96*	3.24×10^{-1}
Portugal	9.10×10^{-1}	3.70×10^{-1}
Romania	0.96*	3.24×10^{-1}
Croatia	9.40×10^{-1}	6.03×10^{-1}
Slovakia	0.96*	3.80×10^{-1}
Slovenia	0.96*	3.24×10^{-1}
Sweden	0.96*	3.24×10^{-1}

*Rest of the World (RoW) dataset was used due to the activity is not available for the particular location.

Supplementary Table 21. Life cycle emission of fossil-based technologies with CCS ($\mathrm{EM}_{j,i}^{\mathrm{Elec}}$) [$kgCO_2/kWh$]

Country	Coal+ CCS	Natural Gas+ CCS
Austria	2.15×10^{-1}	1.32×10^{-1}
Belgium	2.32×10^{-1}	8.99×10^{-2}
Bulgaria	4.15×10^{-1}	8.19×10^{-2}
Cyprus	1.81×10^{-1}	6.53×10^{-2}
Czechia	2.70×10^{-1}	1.18×10^{-1}
Germany	2.33×10^{-1}	9.76×10^{-2}
Denmark	1.81×10^{-1}	7.52×10^{-2}
Spain	3.10×10^{-1}	1.23×10^{-1}
Estonia	2.46×10^{-1}	7.52×10^{-2}
Finland	2.25×10^{-1}	1.99×10^{-1}
France	2.31×10^{-1}	1.17×10^{-1}
United Kingdom	2.39×10^{-1}	6.08×10^{-2}
Greece	1.81×10^{-1}	1.71×10^{-1}
Hungary	1.81×10^{-1}	1.66×10^{-1}
Ireland	2.31×10^{-1}	5.91×10^{-2}
Italy	2.36×10^{-1}	1.13×10^{-1}
Lithuania	1.81×10^{-1}	7.52×10^{-2}
Luxembourg	1.81×10^{-1}	8.19×10^{-2}
Latvia	2.10×10^{-1}	8.19×10^{-2}

Malta	1.81×10^{-1}	8.19×10^{-2}
Netherlands	2.22×10^{-1}	7.27×10^{-2}
Poland	1.81×10^{-1}	1.06×10^{-1}
Portugal	2.28×10^{-1}	9.29×10^{-2}
Romania	1.81×10^{-1}	8.19×10^{-2}
Croatia	2.49×10^{-1}	1.52×10^{-1}
Slovakia	1.81×10^{-1}	1.48×10^{-1}
Slovenia	1.81×10^{-1}	8.19×10^{-2}
Sweden	1.81×10^{-1}	7.28×10^{-2}

Supplementary Table 22 shows the emissions of transporting natural gas (EM^{NGT}), which were sourced from the Ecoinvent activity "market for transport, pipeline, long-distance, natural gas {RER}"[5], together with the life cycle emissions associated with the transportation and injection of CO_2 (EM^{STO}), which were modeled from Wildbolz[20].

Supplementary Table 22. Life cycle emissions associated with the transportation and injection of CO_2 (EM^{STO}) and the natural transportation via pipeline (EM^{NGT}).

**Parameter**	**Value**
EM^{STO}	8.15×10^{-6} tCO_2/tkm
EM^{NGT}	5.24×10^{-2} $kgCO_2$/tkm

*tkm is the abbreviation of ton-kilometer which is a unit representing the transport of one ton of goods over one kilometer with a particular transportation media.

For the biomass-based power technologies, i.e., bioenergy and BECCS, we used the generic supply chain shown in **Supplementary Fig. 1**.

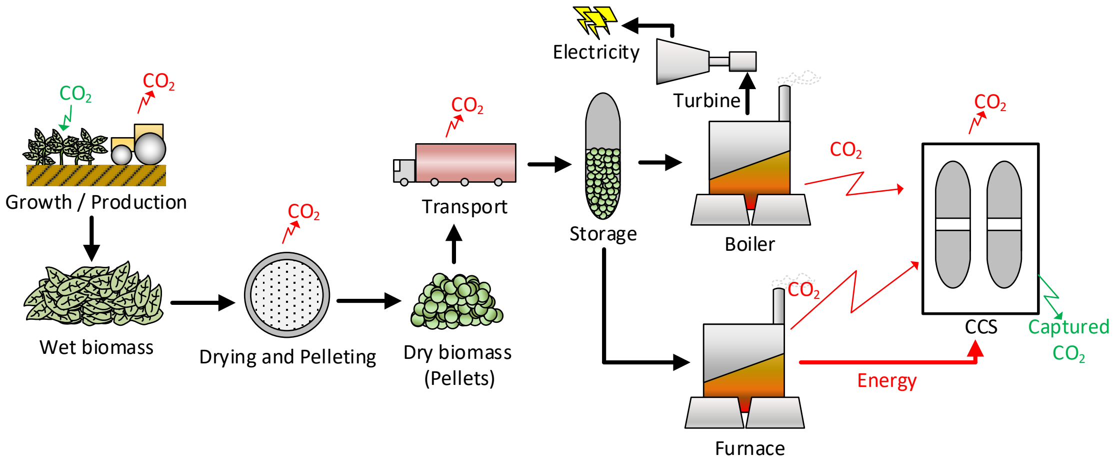

Supplementary Fig. 1. ***Bioenergy with carbon capture and storage supply chain.*** *The biomass without carbon capture and storage (CCS) supply chain is analogous but lacks the furnace and the CCS unit.*

We assume that forestry and agricultural residues have zero emissions embodied (only their carbon content is considered). The emissions from the cultivation of the dedicated bioenergy crops were obtained from the Farm Energy Analysis Tool (FEAT)[21] and regionalized with the yield shown in ***Supplementary Table 30***. The results provided by the FEAT database are expressed as CO_2-eq emissions associated with the fertilizer, lime, seed, herbicide, insecticide, fuel, and

transportation requirements associated with the growth of the specific crops. These data are shown in **Supplementary Table 23**.

Supplementary Table 23. Emissions production for different countries and crops ($EM^{BP}_{j,b}$) [kg CO_2/kg (wb)]

Country	Miscanthus	Switchgrass	Willow
Austria	$3.41x10^{-2}$	$1.07x10^{-1}$	$4.25x10^{-2}$
Belgium	$4.15x10^{-2}$	$8.85x10^{-2}$	$4.49x10^{-2}$
Bulgaria	$4.65x10^{-2}$	$1.07x10^{-1}$	$4.76x10^{-2}$
Cyprus	$3.29x10^{-2}$	$2.74x10^{-1}$	$6.55x10^{-2}$
Czechia	$3.50x10^{-2}$	$1.07x10^{-1}$	$3.07x10^{-2}$
Germany	$4.62x10^{-2}$	$1.07x10^{-1}$	$4.44x10^{-2}$
Denmark	$5.00x10^{-2}$	$1.21x10^{-1}$	$4.99x10^{-2}$
Spain	$2.77x10^{-2}$	$2.14x10^{-1}$	$4.99x10^{-2}$
Estonia	$4.65x10^{-2}$	$2.03x10^{-1}$	$7.99x10^{-2}$
Finland	$3.89x10^{-2}$	$2.74x10^{-1}$	$7.99x10^{-2}$
France	$4.43x10^{-2}$	$9.83x10^{-2}$	$4.54x10^{-2}$
United Kingdom	$5.19x10^{-2}$	$1.07x10^{-1}$	$4.54x10^{-2}$
Greece	$2.13x10^{-2}$	$2.74x10^{-1}$	$4.00x10^{-2}$
Hungary	$4.65x10^{-2}$	$8.85x10^{-2}$	$4.99x10^{-2}$
Ireland	$4.58x10^{-2}$	$1.63x10^{-1}$	$4.65x10^{-2}$
Italy	$2.59x10^{-2}$	$8.41x10^{-2}$	$1.33x10^{-1}$
Lithuania	$4.65x10^{-2}$	$1.07x10^{-1}$	$4.44x10^{-2}$
Luxembourg	$3.69x10^{-2}$	$8.85x10^{-2}$	$4.54x10^{-2}$
Latvia	$4.65x10^{-2}$	$1.63x10^{-1}$	$7.99x10^{-2}$
Malta	$3.29x10^{-2}$	$1.07x10^{-1}$	$6.55x10^{-2}$
Netherlands	$4.43x10^{-2}$	$1.05x10^{-1}$	$4.49x10^{-2}$
Poland	$4.43x10^{-2}$	$1.07x10^{-1}$	$4.99x10^{-2}$
Portugal	$3.32x10^{-2}$	$1.07x10^{-1}$	$4.00x10^{-1}$
Romania	$4.15x10^{-2}$	$1.07x10^{-1}$	$4.99x10^{-2}$
Croatia	$3.69x10^{-2}$	$1.07x10^{-1}$	$3.63x10^{-2}$
Slovakia	$4.15x10^{-2}$	$1.07x10^{-1}$	$5.71x10^{-2}$
Slovenia	$4.15x10^{-2}$	$1.07x10^{-1}$	$4.00x10^{-2}$
Sweden	$4.10x10^{-2}$	$4.97x10^{-1}$	$9.99x10^{-2}$

After the energy crops are harvested, the biomass feedstock is converted into pellets to facilitate its transportation to the power plants. The emissions associated with the drying and pelleting are obtained from the Ecoinvent activity “Wood Pellet Production” for RER (Europe). Moreover, for biomass transportation, we assume that the pellets are transported by lorry, i.e., activity “transport, freight, lorry > 32 metric ton, EURO3, RER, market for transport”. These two parameters are shown in **Supplementary Table 24**.

Supplementary Table 24. Life cycle emissions of the pelletizing process (EM^{BPe}) and biomass transportation emissions via truck (EM^{BT})[5].

Parameter	Value
EM^{BPe}	9.36×10^{-2} $kgCO_2$/kg (db)
EM^{BT}	8.90×10^{-5} tCO_2/tkm

*tkm is the abbreviation of ton-kilometer which is a unit representing the transport of one ton of goods over one kilometer with a particular transportation media.

Similarly, as with the fossil-fueled power plants with CCS, we assume a conservative CO_2 capture rate of 90% considering the post-combustion capture technology with monoethanolamine (MEA) in the BECCS power plants[13]. Note that in the case of biomass power plants without CCS, the biogenic emissions are set to zero, assuming carbon neutrality, as explained before.

In contrast, for BECCS, we account for the CO_2 embodied in the biomass feedstock ($\mathrm{BREM_b}$) modeled as a negative CO_2 input in the system. These emissions can be obtained from the carbon content of the different biomass types ($\mathrm{CC_b}$) and the molecular weights of CO_2 and carbon ($\mathrm{MW^{CO_2}}$ and $\mathrm{MW^{C}}$, respectively) as shown in Eq. 56.

$$\mathrm{BREM_b} = \mathrm{CC_b} \cdot \frac{\mathrm{MW^{CO_2}}}{\mathrm{MW^{C}}} \qquad \forall b \in B \qquad \textit{Eq. 56}$$

The carbon and moisture contents and the Higher Heating Value of the biomass types are obtained from the Phyllis2 database[22] (**Supplementary Table 25**).

Supplementary Table 25. Biomass parameters: Carbon content (wb) ($\mathrm{CC_b}$, expressed in %), humidity ($\mathrm{HUM_b}$, expressed in %) and higher heating value ($\mathrm{HHV_b}$, in MJ/kg(db)).

**Biomass**	$\mathbf{CC_b}$	$\mathbf{HUM_b}$	$\mathbf{HHV_b}$
Miscanthus	28.75	40.00	18.57
Switchgrass	37.06	11.90	16.17
Willow	24.85	50.10	19.75
Straw residues	40.88	9.19	17.85
Agricultural prunings	47.05	4.80	19.57
Forestry residues	47.05	4.80	19.57

The amount of biogenic CO_2 released to the atmosphere (not captured) in the combustion process of the pellets is shown in **Supplementary Table 26**. The amount of carbon released by the biomass matches the amount captured during its growth; hence, for convenience, we modify the basis from wet biomass to dry biomass as follows:

$$\mathrm{EM_b^{BBio}} = \frac{\mathrm{BREM_b}}{(1 - \mathrm{HUM_b})} \qquad \forall b \in B \qquad \textit{Eq. 57}$$

$$\mathrm{EM_b^{BBECCS}} = (1 - \mathrm{HCAP}) \frac{\mathrm{BREM_b}}{(1 - \mathrm{HUM_b})} \qquad \forall b \in B \qquad \textit{Eq. 58}$$

For the CCS case, the direct emissions are calculated considering the capture efficiency parameter (HCAP), equal to 90%, assuming a conservative estimate.

Supplementary Table 26. Direct emissions from burning pellets for different biomass types b ($\mathrm{EM_b^{BBio}}$ and $\mathrm{EM_b^{BBECCS}}$)[kgCO_2/kg (db)].

**Biomass**	$\mathbf{EM_b^{BBio}}$	$\mathbf{EM_b^{BBECCS}}$
Miscanthus	1.76	0.18
Switchgrass	1.58	0.16
Willow	1.83	0.18
Straw residues	1.65	0.17

Agricultural prunings	1.81	0.18
Forestry residues	1.81	0.18

We also performed a sensitivity analysis of the emissions parameters retrieved from Ecoinvent v3.5,[5] which are affected by various uncertainty sources[23]. Accordingly, we used the Simapro v9.0 software[24] to generate 1,000 scenarios via Monte Carlo sampling, considering the default uncertainty data therein (i.e., parameters of the underlying probability distributions of the uncertain emissions). We then defined the optimistic and pessimistic scenarios considering ± 2 times the standard deviation of the samples. (**Supplementary Table 27**).

Supplementary Table 27. Standard deviation of different emissions parameters.

Parameter	Value
Life cycle emissions Wind onshore	1.72×10^{-3} $kgCO_2/kWh$
Life cycle emissions Wind offshore	1.64×10^{-3} $kgCO_2/kWh$
Life cycle emissions Hydro run-of-river	1.88×10^{-3} $kgCO_2/kWh$
Life cycle emissions Hydro reservoir	1.88×10^{-3} $kgCO_2/kWh$
Life cycle emissions Geothermal	31.1×10^{-3} $kgCO_2/kWh$
Life cycle emissions Solar PV open ground	12.8×10^{-3} $kgCO_2/kWh$
Life cycle emissions Solar PV roof	1.39×10^{-3} $kgCO_2/kWh$
Life cycle emissions Solar Thermal	5.68×10^{-3} $kgCO_2/kWh$
Life cycle emissions Coal	139×10^{-3} $kgCO_2/kWh$
Life cycle emissions Natural gas	9.65×10^{-3} $kgCO_2/kWh$
Life cycle emissions Nuclear	2.35×10^{-3} $kgCO_2/kWh$
Life cycle emissions of pelletizing process	5.79×10^{-3} $kgCO_2/kg$ (db)
Life cycle emissions for pellets transportion	8.14×10^{-6} tCO_2/tkm

2.2.5. Biomass parameters

The uptake of CO_2 by the plant via photosynthesis during its growth is calculated with Eq. 56 and shown in **Supplementary Table 28**.

Supplementary Table 28. CO_2 removal via photosynthesis for different types of biomass ($\mathrm{BREM_b}$) [kg CO_2/kg (wb)].

Biomass	$\mathbf{BREM_b}$
Miscanthus	1.05
Switchgrass	1.40
Willow	0.91
Straw residues	1.50
Agricultural prunings	1.73
Forest residues	1.73

The electricity delivered with the bioenergy technologies is calculated from the efficiencies of the boiler and turbine and the HHV of the biomass (Eq. 59). The assumed efficiencies at biomass-based power plants are 72.83 % for the boiler[25]($\mathrm{EFF^{Boiler}}$) and 31.23 % for the turbine[13] ($\mathrm{EFF^{Turbine}}$). The BECCS processes incur an energy (and efficiency) penalty due to the heat required to desorb the CO_2 from the MEA ($\mathrm{HEAT^{MEA}}$, 0.884 kWh/kg of captured CO_2), and the extra electricity needed to operate the CCS system ($\mathrm{ELEC^{Plant}}$, 0.145 kWh/kg of CO_2), mostly needed to compress the captured CO_2[13]. The electricity conversion efficiency parameters (expressed as kWh per kg of pellets combusted) for both Biomass w/o CCS and BECCS plants

($\mathrm{BVAL}_b^{\mathrm{Biomass}}$ and $\mathrm{BVAL}_b^{\mathrm{BECCS}}$, respectively) are displayed in **Supplementary Table 29**, while the associated calculations are explained in detail next.

For the Biomass w/o CCS, the electricity conversion efficiency ($\mathrm{BVAL}_b^{\mathrm{Biomass}}$, expressed in kWh per kg of dry biomass combusted) is calculated from the energy content of the biomass (HHV_b) and the efficiencies of the boiler and the turbine ($\mathrm{EFF}^{\mathrm{Boiler}}$ and $\mathrm{EFF}^{\mathrm{Turbine}}$, respectively) using Eq. 59.

$$\mathrm{BVAL}_b^{\mathrm{Biomass}} = \mathrm{HHV}_b \cdot \mathrm{EFF}^{\mathrm{Boiler}} \cdot \mathrm{EFF}^{\mathrm{Turbine}} \qquad \forall b \in B \qquad \textit{Eq. 59}$$

For the BECCS plants, we consider that one portion of the biomass input will be combusted to cover the heating needs of the desorption process ($\mathrm{Biomass}_b^{\mathrm{Heating}}$), while the rest is used to generate the electricity required to operate the CCS system (electricity penalty).

The heating required to regenerate the MEA solution (kg for heating per kg of biomass input, $\mathrm{Biomass}_b^{\mathrm{Heating}}$) is calculated in Eq. 60 considering the heating needs of the MEA per mass of CO_2 captured ($\mathrm{HEAT}^{\mathrm{MEA}}$). The amount of CO_2 captured is determined from the capture rate (HCAP) and the CO_2 embodied in the biomass entry (carbon uptake via photosynthesis, BREM_b), which is released during biomass combustion. Finally, the inverse of the higher heating value of the biomass (HHV_b) provides the amount of biomass required to cover the said heating needs.

$$\mathrm{Biomass}_b^{\mathrm{Heating}} = \mathrm{HEAT}^{\mathrm{MEA}}\ \mathrm{HCAP}\ \frac{\mathrm{BREM}_b}{(1-\mathrm{HUM}_b)}\ \frac{1}{\mathrm{HHV}_b} \qquad \forall b \in B \qquad \textit{Eq. 60}$$

The remaining fraction of the biomass is used to generate electricity ($\mathrm{Biomass}_b^{\mathrm{Electricity}}$, expressed in kg of biomass providing electricity per kg of biomass input), as shown in Eq. 61.

$$\mathrm{Biomass}_b^{\mathrm{Electricity}} = 1 - \mathrm{Biomass}_b^{\mathrm{Heating}} \qquad \forall b \in B \qquad \textit{Eq. 61}$$

Finally, the value of BVAL_b for BECCS is obtained considering the power produced from the fraction of biomass used for electricity generation ($\mathrm{Biomass}_b^{\mathrm{Electricity}}$), its higher heating value (HHV_b), the efficiencies of the boiler and turbine ($\mathrm{EFF}^{\mathrm{Boiler}}$ ·and $\mathrm{EFF}^{\mathrm{Turbine}}$), and an electricity penalty per kg of CO_2 captured (estimated considering the parameter $\mathrm{ELEC}^{\mathrm{Plant}}$).

$$\mathrm{BVAL}_b^{\mathrm{BECCS}} = \mathrm{Biomass}_b^{\mathrm{Electricity}} \mathrm{HHV}_b \cdot \mathrm{EFF}^{\mathrm{Boiler}} \cdot \mathrm{EFF}^{\mathrm{Turbine}} - \mathrm{ELEC}^{\mathrm{Plant}} \mathrm{HCAP}\ \frac{\mathrm{BREM}_b}{(1-\mathrm{HUM}_b)} \qquad \forall b \in B \qquad \textit{Eq. 62}$$

Supplementary Table 29. Electricity conversion efficiency for different biomass types *b* in bot biomass w/o CCs ($\mathrm{BVAL}_b^{\mathrm{Biomass}}$) and BECCS plants ($\mathrm{BVAL}_b^{\mathrm{BECCS}}$) [kWh/kg (db)].

**Biomass**	$\mathbf{BVAL_b^{Biomass}}$	$\mathbf{BVAL_b^{BECCS}}$
Miscanthus	1.17	0.63
Switchgrass	1.02	0.53
Willow	1.25	0.68
Straw residues	1.13	0.61
Agricultural prunings	1.24	0.67
Forest residues	1.24	0.67

The yields of the bioenergy crops were sourced from Fajardy et al.[26].

Supplementary Table 30. Biomass yield per energy crop and country ($PY_{j,b}$) [t/ha/yr (db)].

Country	Miscanthus	Switchgrass	Willow
Austria	19.50	8.00	9.40
Belgium	16.00	12.00	8.90
Bulgaria	14.30	8.00	8.40
Cyprus	20.20	2.00	6.10
Czechia	19.00	8.00	13.00
Germany	14.40	8.00	9.00
Denmark	13.30	7.10	8.00
Spain	24.00	4.00	8.00
Estonia	14.30	4.00	5.00
Finland	17.10	2.00	5.00
France	15.00	9.50	8.80
United Kingdom	12.80	8.00	8.80
Greece	31.20	2.00	10.00
Hungary	14.30	12.00	8.00
Ireland	14.50	4.00	8.60
Italy	25.70	13.60	3.00
Lithuania	14.30	8.00	9.00
Luxembourg	18.00	12.00	8.80
Latvia	14.30	4.00	5.00
Malta	20.20	8.00	6.10
Netherlands	15.00	8.30	8.90
Poland	15.00	8.00	8.00
Portugal	20.00	8.00	1.00
Romania	16.00	8.00	8.00
Croatia	18.00	8.00	11.00
Slovakia	16.00	8.00	7.00
Slovenia	16.00	8.00	10.00
Sweden	16.20	1.00	4.00

2.2.6. Storage parameters

The amount of CO_2 captured post-combustion using monoethanolamine (MEA) solvent at the coal and natural gas power plants, which is stored in geological reservoirs (**Supplementary Table 31**), was calculated assuming a CO_2 capture rate of 90%, and a surplus of fuel –to cover the energy requirements of the CCS system– of 31.2% and 16.3% for coal and natural gas, respectively [8].

Supplementary Table 31. CO_2 post-combustion captured in Coal and Natural Gas with CCS power plants ($STO_{j,i}^{Elec}$) [kg CO_2/kWh].

Country	Coal + CCS	Natural Gas + CCS
Austria	0.95	0.38
Belgium	1.11	0.35
Bulgaria	1.92	0.34
Cyprus	1.13	0.34

Czechia	1.18	0.38
Germany	1.05	0.36
Denmark	1.13	0.41
Spain	1.10	0.40
Estonia	1.13	0.41
Finland	1.02	0.65
France	1.08	0.48
United Kingdom	1.06	0.34
Greece	1.13	0.43
Hungary	1.13	0.43
Ireland	1.02	0.37
Italy	1.08	0.38
Lithuania	1.13	0.41
Luxembourg	1.13	0.34
Latvia	0.97	0.34
Malta	1.13	0.34
Netherlands	1.00	0.34
Poland	1.13	0.34
Portugal	1.08	0.39
Romania	1.13	0.34
Croatia	1.11	0.64
Slovakia	1.13	0.40
Slovenia	1.13	0.34
Sweden	1.13	0.34

In the case of BECCS, the amount of CO_2 captured at the power plant and sent to storage is calculated considering that 90% of the direct CO_2 emissions from the combustion of the pellets are captured (**Supplementary Table 32**).

Supplementary Table 32. CO_2 post-combustion captured for BECCS (STO_i^B) [kgCO_2/kg (db)]

**Biomass**	$\mathbf{STO_i^B}$
Miscanthus + CCS	1.58
Switchgrass + CCS	1.43
Willow + CCS	1.64
Straw residues + CCS	1.49
Agricultural prunings + CCS	1.63
Forest residues + CCS	1.63

The capacity available for CO_2 storage in the EU countries was sourced from the EU GeoCapacity project[27], which considers potentials for deep saline aquifers, hydrocarbon fields, and coals fields (except for Sweden and Finland, which did not participate in the EU GeoCapacity project[27]). Finland has no suitable underground fields for CO_2 long-term storage, while for Sweden, the geological capacity was sourced from Mortensen et al.[28]. Data on geological capacity in each country are summarized in **Supplementary Table 33**.

Supplementary Table 33. CO_2 geological storage capacity for different countries (STO_j^{Cap}) [GtCO_2].

**Country**	STO_j^{Cap}

Austria	0.00
Belgium	0.20
Bulgaria	2.12
Cyprus	0.00
Czechia	0.85
Germany	17.08
Denmark	2.76
Spain	14.18
Estonia	0.00
Finland	0.00
France	8.69
United Kingdom	14.40
Greece	0.25
Hungary	0.62
Ireland	0.00
Italy	6.55
Lithuania	0.04
Luxembourg	0.00
Latvia	0.40
Malta	0.00
Netherlands	2.34
Poland	2.94
Portugal	0.00
Romania	9.00
Croatia	2.90
Slovakia	1.72
Slovenia	0.09
Sweden	3.40

2.2.7. Demand parameters

The electricity demand data in each country for 2020 was obtained from the EU statistical pocketbook[29] (**Supplementary Table 34**).

Supplementary Table 34. Electricity demand in European countries for 2020 ($D_{j,t}^{Elec}$) [Mtoe/yr].

**Country**	$D_{j,t}^{Elec}$
Austria	5.40
Belgium	7.04
Bulgaria	2.57
Cyprus	0.39
Czechia	4.93
Germany	44.62
Denmark	2.69
Spain	20.17
Estonia	0.62
Finland	6.97
France	37.56
United Kingdom	25.85
Greece	4.64
Hungary	3.31
Ireland	2.22
Italy	25.10

Lithuania	0.86
Luxembourg	0.55
Latvia	0.56
Malta	0.20
Netherlands	9.08
Poland	11.68
Portugal	4.01
Romania	3.84
Croatia	1.37
Slovakia	2.22
Slovenia	1.16
Sweden	10.94

The future electricity demand was estimated based on historical data and projections[30]. In particular, we consider an expected growth in electricity demand in 2000-2050 from 3000 TWh to approximately 4250 TWh. Notably, RAPID considers a constant annual growth until 2100, resulting in a yearly increment of 0.7 %.

2.2.8. Resources potential: limit parameters

The maximum amount of heat generated in each country is limited by the primary production of natural gas energy in 2017, shown in **Supplementary Table 35**[29]. Unlimited availability of natural gas is assumed for Russia.

Supplementary Table 35. Upper bound on the heat from natural gas generated in each country ($\mathrm{LIM}_j^{\mathrm{Heat}}$) [Mtoe/yr].

**Country**	$\mathrm{LIM}_j^{\mathrm{Heat}}$
Austria	1.04
Belgium	0
Bulgaria	0.07
Cyprus	0.19
Czechia	0
Germany	6.03
Denmark	4.35
Spain	0.02
Estonia	0
Finland	0
France	0.01
United Kingdom	36.02
Greece	0.01
Hungary	1.41
Ireland	2.85
Italy	4.54
Lithuania	0
Luxembourg	0
Latvia	0
Malta	0
Netherlands	33.17
Poland	3.51
Portugal	0

Romania	8.52
Croatia	1.23
Slovakia	0.12
Slovenia	0.01
Sweden	0
Russia	∞

For the renewable technologies, the following data on potentials were considered (**Supplementary Table 36**).

Data for wind onshore and offshore, solar PV open ground and rooftop installations, and concentrated solar power technologies were sourced from the ENSPRESO database aggregated at the country level[31].

For wind onshore, we considered wind conditions with capacity factors above 25% and a high level of exclusion of surfaces for wind (EU-Wide high restrictions). Moreover, we considered wind offshore potentials in water depth on 0-30 m, 30-60 m, with any wind conditions and EU-Wide high restrictions.

For Solar PV open ground, we considered a potential of 85 MW/km^2 (south orientation 45%) and only non-artificial areas, assuming that 20% of the agriculture low irradiation areas and 100% of natural non-agriculture low irradiation areas are available. For solar PV rooftop, we included both residential and industrial areas regardless of the facade orientation (north, south, east, west) and roof-top inclination. For Concentrated Solar Power, which competes with Solar PV ground-mounted for the land available, we considered a potential of 85 MW/km^2 and 100% of the available non-artificial areas with high irradiation. Note that solar PV considers low irradiation areas and, therefore, its potential does not overlap with that of CSP power. Data for hydropower technologies (run-of-river and reservoir) were sourced from e-highways[32] "Energy production in Europe by country in 2050 – 100% RES". The geothermal data were taken from the literature [29,33–37].

Supplementary Table 36. Potential electricity production for the renewable technologies by country ($GEN_{j,i}^{Pot}$) [TWh].

**Country**	**Wind onshore**	**Wind offshore**	**Hydro run-of-river**	**Hydro reservoir**
Austria	45.24	0.00	43.86	11.39
Belgium	0.30	0.00	1.77	0.00
Bulgaria	7.75	0.00	5.75	8.93
Cyprus	0.00	0.00		0.00
Czechia	13.43	0.00	2.10	1.31
Germany	57.14	4.00	24.67	0.00
Denmark	40.12	0.00	0.07	0.00
Spain	600.70	0.00	37.67	26.07
Estonia	43.05	1.00	0.33	0.00
Finland	42.32	54.00	8.88	7.81
France	423.29	12.00	56.66	33.45
UnitedKingdom	502.53	187.00	4.62	12.92
Greece	254.76	0.00	3.62	15.87
Hungary	22.33	0.00	4.61	0.00
Ireland	277.34	0.00	1.07	0.00
Italy	117.52	2.00	25.94	33.38

Lithuania	129.19	1.00	1.20	0.00
Luxembourg	0.00	0.00	0.94	0.00
Latvia	99.00	19.00	3.99	0.00
Malta	0.00	0.00		0.00
Netherlands	9.91	0.00	0.75	0.00
Poland	224.01	1.00	12.02	0.00
Portugal	7.00	0.00	14.40	9.50
Romania	38.54	1.00	29.69	9.98
Croatia	9.14	1.00	3.22	8.57
Slovakia	11.26	0.00	6.55	0.00
Slovenia	0.28	0.00	8.82	0.00
Sweden	301.36	42.00	13.93	82.92

**Country**	**Geothermal**	**Solar Photovoltaic open ground**	**Solar Photovoltaic roof**	**Solar Thermal Parabolic**
Austria	0.00	422.94	9.95	0.00
Belgium	0.00	344.84	12.31	0.00
Bulgaria	0.00	1,291.77	10.60	0.00
Cyprus	0.00	275.30	1.67	205.98
Czechia	3.00	655.55	11.76	0.00
Germany	1.00	3,247.60	86.60	0.00
Denmark	0.00	462.56	6.18	0.00
Spain	1.00	3,389.62	63.00	10,539.06
Estonia	0.00	145.00	1.36	0.00
Finland	0.00	159.60	5.57	0.00
France	0.00	6,388.85	82.16	270.23
United Kingdom	0.00	1,666.42	62.13	0.00
Greece	0.00	145.00	16.67	219.23
Hungary	17.00	159.60	13.26	0.00
Ireland	0.00	6,388.85	4.53	0.00
Italy	12.00	3,972.01	83.62	437.21
Lithuania	0.00	813.00	3.19	0.00
Luxembourg	0.00	14.45	0.60	0.00
Latvia	0.00	260.77	2.10	0.00
Malta	0.00	2.06	0.71	5.49
Netherlands	0.00	373.06	17.38	0.00
Poland	0.00	2,617.80	41.68	0.00
Portugal	0.20	372.91	11.87	1,264.89
Romania	0.00	2,659.00	26.75	0.00
Croatia	3.00	525.03	5.95	1.08
Slovakia	1.00	354.10	6.57	0.00
Slovenia	0.00	108.63	2.59	0.00
Sweden	0.00	328.67	9.78	0.00

The estimates for the marginal land area available for growing energy crops were sourced from Pozo et al. (2020)[38]. The authors followed a conservative approach, using the original estimates from Cai et al.[39] based on the most conservative scenario (i.e., scenario S1), which accounts for soil productivity, slope, climate, and land cover conservative criteria. Then, following Fritz et al.[40], these land estimates at the country level were further downgraded by 69%, leading to even

more conservative data. Our estimates for marginal land available include at least part of the abandoned, wasted, or idle agricultural land and some small crop fields, which alleviate issues related to land competition with food production and other sustainability concerns. Nevertheless, the marginal land available is highly uncertain. Sectorial competition for the limited marginal land might arise in the future, reducing the land available for energy purposes. Other authors argue that more land might eventually become available due to improvements in agriculture or dietary changes[41]. Hence, to understand how uncertainties in the marginal land available affect our results, we performed a sensitivity analysis considering different scenarios with increased/reduced land available (details in the Methods section and results in **Supplementary Fig. 2**).

We note that the nuclear power capacity cannot increase with time (Supplementary Table 39) because we do not contemplate installing additional facilities. We adopted this assumption based on the recent emergence of phase-out plans for coal and nuclear power in Europe (e.g., nuclear in Germany, Belgium, or Switzerland)[42,43]. The capacity limit for coal and natural gas power is twice the current installed capacity (Supplementary Table 39).

Supplementary Table 37. Limit on the capacity of coal, natural gas ($\mathrm{LIM_j^{NG}}$ and $\mathrm{LIM_j^{Coal}}$), and nuclear power plants ($\mathrm{LIM_j^{Nuclear}}$) [MW] and area available ($\mathrm{LIM_j^{Area}}$) [ha] in each country.

**Country**	$\mathbf{LIM_j^{NG}}$	$\mathbf{LIM_j^{Coal}}$	$\mathbf{LIM_j^{Nuclear}}$	$\mathbf{LIM_j^{Area}}$
Austria	8,030	492	0	39,796
Belgium	13,298	940	5,931	17,889
Bulgaria	2,464	8,950	2,000	166,994
Cyprus	1,478	1,478	0	952
Czechia	2,452	20,060	4,040	66,497
Germany	63,328	92,996	9,516	211,107
Denmark	3,628	7,312	0	25,962
Spain	60,532	19,122	7,117	2,127,822
Estonia	250	102	0	352,613
Finland	3,824	4,556	2,785	1,785
France	23,904	7,932	63,130	298,899
United Kingdom	75,396	17,588	9,229	480,510
Greece	9,804	7,824	0	150,344
Hungary	8,056	2,098	1,899	53,865
Ireland	8,530	1,710	0	473,295
Italy	92,644	13,326	0	156,449
Lithuania	3,420	0	0	493,481
Luxembourg	162	0	0	371
Latvia	2,220	0	0	436,784
Malta	1,076	0	0	0
Netherlands	31,140	9,262	486	28,692
Poland	4,212	61,092	0	737,129
Portugal	9,212	3,512	0	381,688
Romania	6,066	8,256	1,300	201,652
Croatia	1,486	664	0	52,259
Slovakia	2,222	1,132	1,940	48,858
Slovenia	982	1,848	696	10,510
Sweden	0	0	8,586	141,918

The residues available by country and per year are shown in **Supplementary Table 38**. The estimates for straw residues, agricultural prunings, and forestry residues implicitly consider sustainable practices (e.g., soil conservation and biodiversity protection). Moreover, the estimates discount other competitive uses of such residues (e.g., straw for animal bedding or prunings for composting and firewood)[44–46].

Supplementary Table 38. Residues potential in each country ($\mathrm{LIM}_{\mathrm{j,b}}^{\mathrm{BR}}$) [t/yr (wb)]

Country	Straw residues[44]	Agricultural prunings[45]	Forest residues[46]
Austria	1,941,413	152,247	16,921,420
Belgium	957,802	57,093	2,462,967
Bulgaria	4,003,269	767,580	3,854,125
Cyprus	0	98,326	0
Czechia	4,152,388	31,718	12,151,984
Germany	25,473,524	415,508	50,050,490
Denmark	3,727,973	19,031	1,655,068
Spain	6,174,096	13,207,451	11,746,591
Estonia	817,286	6,344	6,269,738
Finland	1,651,779	25,375	39,072,530
France	31,544,384	3,159,131	39,270,607
United Kingdom	6,062,257	47,577	7,278,024
Greece	1,258,908	2,540,626	2,200,212
Hungary	9,124,930	0	5,263,762
Ireland	157,722	0	1,869,891
Italy	9,190,886	6,556,148	11,961,415
Lithuania	1,651,779	44,405	4,631,995
Luxembourg	0	0	456,212
Latvia	788,610	22,203	7,768,885
Malta	0	0	0
Netherlands	559,196	41,234	680,275
Poland	17,613,237	1,024,497	27,554,623
Portugal	544,858	1,858,685	4,864,143
Romania	9,497,727	995,951	15,798,792
Croatia	1,142,288	318,939	3,644,498
Slovakia	2,371,564	28,546	5,300,721
Slovenia	364,194	63,436	4,119,189
Sweden	1,709,132	69,780	49,762,326

2.2.9. Installed capacity today parameters

The capacity installed in 2020 for each power technology in each country was sourced from Entsoe for 2019, which provides the installed net generation capacity –effectively installed on January 1st of the following year–[47]. For coal, data correspond to the summation of fossil hard coal and fossil brown coal/lignite. Due to data gaps in Entsoe, for Slovakia data correspond to 2018, while for hydropower technologies in the United Kingdom, the data are gathered from Eurostast[48]. For Malta, data on the installed capacity for solar and biomass were sourced from ref,[49] and for natural gas based on the values reported by the Enemalta corporation[50]. For Concentrated Solar Power, missing in the previous reference, the installed capacity was sourced from EurObserver[51]. Due to data gaps, we assume that the age of the facilities in 2020 matches the midpoint of their useful life. For the solar PV technologies (open ground and roof), we divide

the total capacity sourced from Entsoe evenly among the subcategories according to the specific data on capacities provided by the International Energy Agency[52]. Notably, according to the source, there is no power technology with CCS installed today. The data are shown in **Supplementary Table 39**.

Supplementary Table 39. Current capacity installed for each technology *i* in country *j* ($\mathrm{CAP}_{\mathrm{j,i}}^{\mathrm{Today}}$) [MW][47,48].

Country	Wind onshore	Wind offshore	Hydro run-of-river	Hydro reservoir
Austria	3,133	0	5,724	2,436
Belgium	2,248	1,548	181	0
Bulgaria	700	0	537	1,810
Cyprus	158	0	0	0
Czechia	316	0	334	753
Germany	52,792	6,393	3,983	1,298
Denmark	4,426	1,700	7	0
Spain	22,961	0	1,156	19,146
Estonia	462	0	0	0
Finland	2,013	0	3,148	0
France	13,610	0	10,955	8,279
United Kingdom	13,633	0	732	732
Greece	2,355	0	299	2,403
Hungary	327	0	30	28
Ireland	1,919	0	216	0
Italy	9,617	0	10,650	3,857
Lithuania	525	0	128	0
Luxembourg	154	0	25	11
Latvia	59	0	1,539	0
Malta	0	0	0	0
Netherlands	3,669	957	38	0
Poland	5,808	0	435	157
Portugal	5,127	0	2,858	1,515
Romania	2,968	0	2,770	3,373
Croatia	616	0	421	1,436
Slovakia	3	0	1,208	418
Slovenia	3	0	1,053	0
Sweden	7,506	0	0	16,301

Country	Geothermal	Solar photovoltaic open ground	Solar photovoltaic roof	Solar Thermal Parabolic
Austria	0	667	667	0
Belgium	0	1,685	1,685	0
Bulgaria	0	530	530	0
Cyprus	0	75	75	0
Czechia	0	1,025	1,025	0
Germany	42	22,650	22,650	0
Denmark	0	507	507	0
Spain	0	3,376	3,376	2,304
Estonia	0	17	17	0
Finland	0	2	2	0

France	0	4,094	4,094	0
United Kingdom	0	6,719	6,719	0
Greece	0	1,221	1,221	0
Hungary	3	468	468	0
Ireland	17	0	0	0
Italy	869	2,359	2,359	0
Lithuania	0	41	41	0
Luxembourg	0	68	68	0
Latvia	0	0	0	0
Malta	0	77	77	0
Netherlands	0	1,969	1,969	0
Poland	0	215	215	0
Portugal	0	162	162	0
Romania	0	575	575	0
Croatia	10	27	27	0
Slovakia	0	266	266	0
Slovenia	0	138	138	0
Sweden	0	0	0	0

Country	Coal	Natural Gas	Nuclear	Biomass
Austria	246	4,015	0	497
Belgium	470	6,649	5,931	708
Bulgaria	4,475	1,232	2,000	74
Cyprus	739	739	0	12
Czechia	10,030	1,226	4,040	400
Germany	46,498	31,664	9,516	7,752
Denmark	3,656	1,814	0	1,772
Spain	9,561	30,266	7,117	507
Estonia	51	125	0	157
Finland	2,278	1,912	2,785	1,804
France	3,966	11,952	63,130	1,931
United Kingdom	8,794	37,698	9,229	0
Greece	3,912	4,902	0	51
Hungary	1,049	4,028	1,899	246
Ireland	855	4,265	0	0
Italy	6,663	46,322	0	1,538
Lithuania	0	1,710	0	98
Luxembourg	0	81	0	30
Latvia	0	1,110	0	126
Malta	0	537	0	5
Netherlands	4,631	15,570	486	485
Poland	30,546	2,106	0	849
Portugal	1,756	4,606	0	605
Romania	4,128	3,033	1,300	115
Croatia	332	743	0	71
Slovakia	566	1,111	1,940	224
Slovenia	924	491	696	17
Sweden	0	0	8,586	0

The binary parameter that activates today's capacity in a given period is computed as follows:

$$\mathrm{PARCAP}_{\mathrm{i,t}}^{\mathrm{Today}} = 1 \qquad \forall i \in I, t \in T: t \leq [\mathrm{UL_i}/2] \qquad \textit{Eq. 63}$$

$$\mathrm{PARCAP}_{i,t}^{\mathrm{Today}} = 0 \qquad \forall i \in I, t \in T: t > \lceil \mathrm{UL_i}/2 \rceil \qquad \textit{Eq. 64}$$

2.2.10. Other parameters

The capacity factor for the electricity technologies is obtained from Carlsson et al.[7] and shown in **Supplementary Table 40**. The capacity factors for the periods missing in the table are assumed to be the same as those reported.

Supplementary Table 40. Capacity factor of each electricity technology *i* and period *t* ($\mathrm{CF_{i,t}}$) [dimensionless].

Technology	**2020**	**2030**	**2040**	**2050**
Wind onshore	0.30	0.35	0.40	0.45
Wind offshore	0.40	0.46	0.48	0.48
Hydro run-of-river	0.37	0.37	0.37	0.37
Hydro reservoir	0.35	0.35	0.35	0.35
Geothermal	0.95	0.95	0.95	0.95
Solar photovoltaic open ground	0.19	0.19	0.19	0.19
Solar photovoltaic roof	0.19	0.19	0.19	0.19
Solar parabolic thermal	0.38	0.40	0.41	0.41
Coal	0.85	0.85	0.85	0.85
Natural Gas	0.85	0.85	0.85	0.85
Nuclear	0.81	0.81	0.81	0.81
Coal + CCS	0.85	0.85	0.85	0.85
Natural Gas + CCS	0.85	0.85	0.85	0.85
Biomass	0.85	0.85	0.85	0.85
BECCS	0.85	0.85	0.85	0.85

The useful life of each electricity technology is obtained from Child et al.[6] and shown in **Supplementary Table 41**.

Supplementary Table 41. Useful life for each technology ($\mathrm{UL_i}$) [y]

**Technology**	$\mathrm{UL_i}$
Wind	25
Hydro	60
Geothermal	40
Solar	30
Coal	40
Natural Gas	35
Nuclear	40
Biomass	30

The remaining parameters values are shown in **Supplementary Table 42**.

Supplementary Table 42. Other parameters.

**Parameter**	**Value**
DPER	5 y
BUC	0.50[53]
ED	0.50
YH	8760 h/yr

ELOSS	7 % /1000 km[54]
HHV^{NG}	55.25 MJ/kg[55]
CAP^{EF}	0.20
$OPEN^{Tech}$	60 MW
$INITIAL^{BECCS}$	7000 MW
$LOSS^{cul}$	2 %
$LOSS^{pell}$	2 %[16]

The time horizon spans until 2100, and is divided into 16 intervals of five years each.

The capacity diffusion rate is set to 20% per year, which corresponds to the maximum value observed in energy-related technologies[2]. An example of how this diffusion rate affects the maximum capacity is shown in the supplementary results (**Supplementary Fig. 4**)

We assume 60 MW of installed capacity in Europe for all the power technologies that have not been deployed yet. This assumption allows expansions in capacity in those technologies with zero current capacity ($\mathrm{CAP}_{j,i}^{Today}$), for example, Natural gas with CCS. Note that this is a very conservative estimate, since the capacity of a single coal plant can be as high as 300 MW. The initial capacity for BECCS is set to 250 MW in each of the 28 EU countries (i.e., 7,000 MW at the European level), based on the state-of-the-art largest standalone biomass-fired combustion power plants.

3. Supplementary results

3.1. Results uncertainty on costs

We include here the results of the uncertainty analysis on the economic performance, as shown in **Supplementary Table 43-50**. In essence, we ran RAPID for the nominal cost parameters and then re-calculated the objective function considering the lower and upper bounds on the CAPEX expenditures of the technologies.

Supplementary Table 43. Uncertainty results for the equipotential curve of 0 Gt [billion 2015€].

Starting year	Minimum cost	Maximum cost
2020	18,570	26,592
2025	18,542	26,654
2030	18,424	26,745
2035	18,396	26,892
2040	18,343	27,151
2045	18,279	27,515
2050	18,228	27,799
2055	18,240	27,958
2060	18,251	28,104
2065	18,229	28,406
2070	18,236	28,809
2075	18,153	29,540
2080	18,233	31,436

Supplementary Table 44. Uncertainty results for the equipotential curve of -10 Gt [billion 2015€].

Starting year	Minimum cost	Maximum cost
2020	19,027	27,703
2025	18,979	27,863
2030	18,910	27,982
2035	18,843	28,180
2040	18,871	28,452
2045	19,021	28,749
2050	18,988	29,115
2055	18,851	29,491
2060	18,876	29,724
2065	18,709	30,446

Supplementary Table 45. Uncertainty results for the equipotential curve of -20 Gt [billion 2015€].

Starting year	Minimum cost	Maximum cost
2020	19,530	28,965
2025	19,556	29,108
2030	19,570	29,204
2035	19,625	29,437
2040	19,610	29,838
2045	19,678	30,325
2050	19,732	30,759
2055	19,781	31,378
2060	20,060	32,360

Supplementary Table 46. Uncertainty results for the equipotential curve of -30 Gt [billion 2015€].

Starting year	Minimum cost	Maximum cost
2020	20,345	30,162
2025	20,306	30,413
2030	20,253	30,655
2035	20,352	31,052
2040	20,565	31,450
2045	20,692	32,030
2050	20,980	32,577
2055	21,630	34,216

Supplementary Table 47. Uncertainty results for the equipotential curve of -40 Gt [billion 2015€].

Starting year	Minimum cost	Maximum cost
2020	21,028	31,649
2025	21,101	32,056
2030	21,259	32,233
2035	21,517	32,592
2040	21,825	33,006
2045	22,080	33,756
2050	22,817	35,194

Supplementary Table 48. Uncertainty results for the equipotential curve of -50 Gt [billion 2015€].

Starting year	Minimum cost	Maximum cost
2020	22,204	313,147
2025	22,374	33,526
2030	22,555	33,716
2035	22,850	34,110
2040	23,072	34,857
2045	23,398	36,234
2050	25,831	38,769

Supplementary Table 49. Uncertainty results for the equipotential curve of -60 Gt [billion 2015€]

Starting year	Minimum cost	Maximum cost
2020	23,218	34,960
2025	23,411	35,449
2030	23,528	35,815
2035	23,899	36,583
2040	24,711	37,850
2045	25,927	39,745

Supplementary Table 50. Uncertainty results for the equipotential curve of -70 Gt [billion 2015€]

Starting year	Minimum cost	Maximum cost
2020	24,565	39,260
2025	24,942	40,253
2030	25,540	41,722
2035	26,910	44,794

3.2. Results uncertainty on biomass potential

Biomass residues and marginal land availability are highly uncertain, so we carried out a sensitivity analysis to analyze the associated implications. Notably, the inherent versatility of biomass in the transition toward a defossilized economy may lead to sectoral competition for the limited biomass resources available. At the same time, sustainability concerns may result in less marginal land available. Conversely, more land might eventually become available due to improvements in agriculture or dietary changes[41]. **Supplementary Fig. 2** shows the results of varying the biomass resources availability within a given range (i.e., ±10%, ±25%, ±50% of the central estimates shown in Supplementary Tables 37 and 38 for marginal land available and amount of residues, respectively).

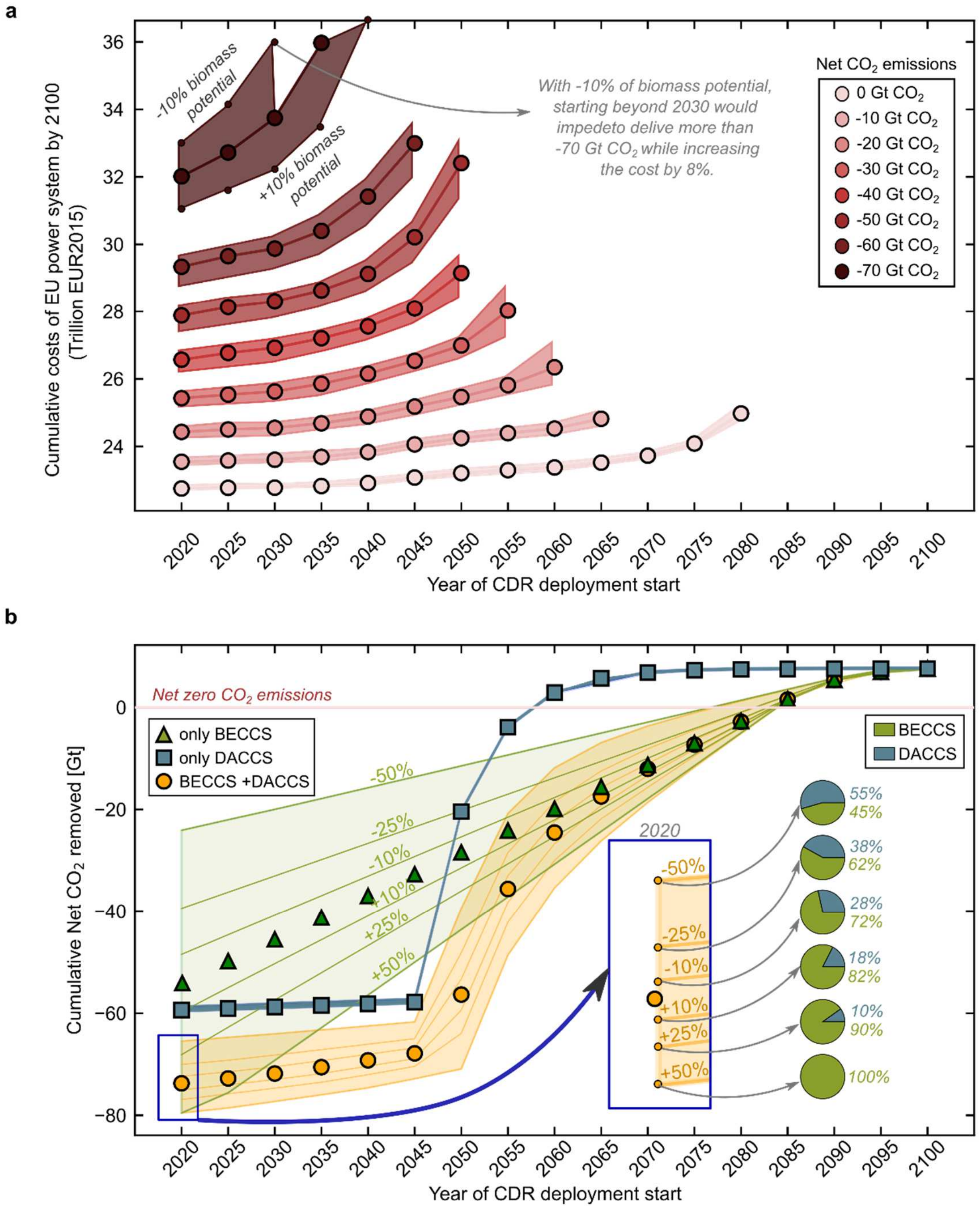

Supplementary Fig. 2. Sensitivity analysis on the biomass potentials (biomass residues and marginal land). *Dots correspond to the optimal solutions deploying bioenergy with carbon capture and storage (BECCS) and direct air carbon capture and storage (DACCS) from a particular point in time onwards (from 2020 to 2100). The shaded areas indicate the new results for a given percentage change in biomass potentials. Subplot* ***a*** *shows the sensitivity analysis for the minimum costs of the European power system associated with increasing carbon dioxide removal (CDR) targets. Subplot* ***b*** *corresponds to the sensitivity analysis for the maximum CDR attainable. In subplot b, the green profile considers only BECCS, blue DACCS, and yellow both BECCS and DACCS, while the pie charts illustrate the proportion of gross CDR provided with BECCS and DACCS, respectively.*

3.3. Results in the context of the EU Green Deal by 2050

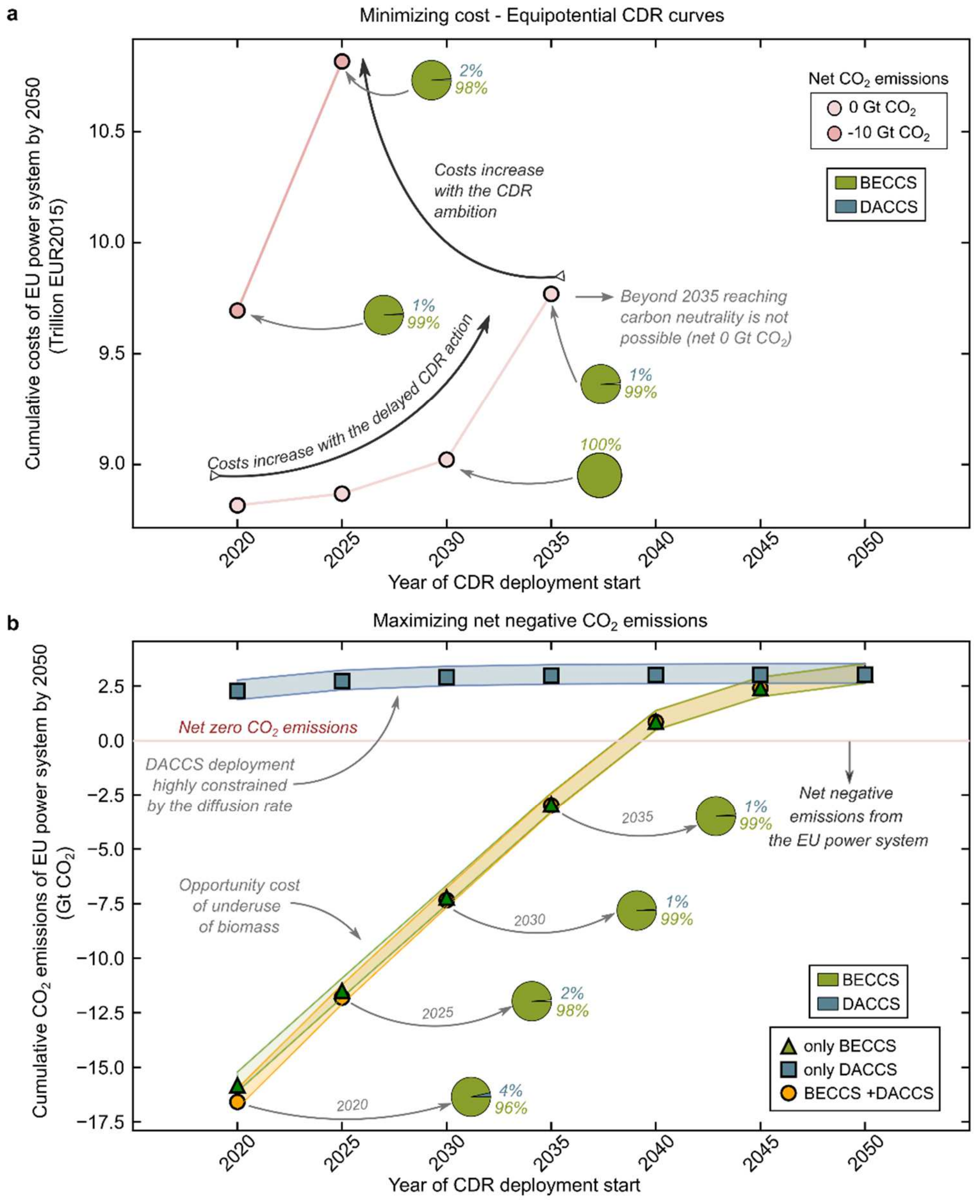

***Supplementary Fig. 3**. Implications on costs and emissions of delayed-actions on carbon dioxide removal (CDR) for different starting points for bioenergy with carbon capture and storage (BECCS) and direct air carbon capture and storage (DACCS) considering the EU climate neutrality goals by 2050 (x-axis).* *Subplot **a** shows the minimum costs of the EU power system associated with increasing CDR targets. Subplot **b** shows the maximum cumulative net CDR that could be attained deploying BECCS and DACCS from a particular point in time onwards (green profile only with BECCS, blue with DACCS, and yellow considering both BECCS and DACCS). Dots correspond to the optimal solutions for the 5-year time steps starting in 2020 and ending in 2020. The shaded areas in subplot **b** indicate the ranges of the results considering the uncertainty in the life cycle CO_2 emissions (i.e., $\mu \pm 2\sigma$, Methods for details on the uncertainty analysis). The pie charts illustrate the proportion of gross CDR provided with BECCS and DACCS, respectively.*

3.4. Maximum technology deployment rate

The maximum deployment rate of technologies, known as diffusion rate, establishes the maximum speed at which technologies can be deployed considering the capacity already installed. **Supplementary Fig. 4** shows an example of the time required to achieve 1 TW of installed capacity for the power technologies and 200 MtCO_2/yr with DACCS considering their given initial power capacities (2020) and a 20% diffusion rate, which has been observed in other energy-related technologies[2]. For the DACCS the initial capacity is set to 1 Mt of gross CO_2 captured per year —reflecting the current scale ambition.

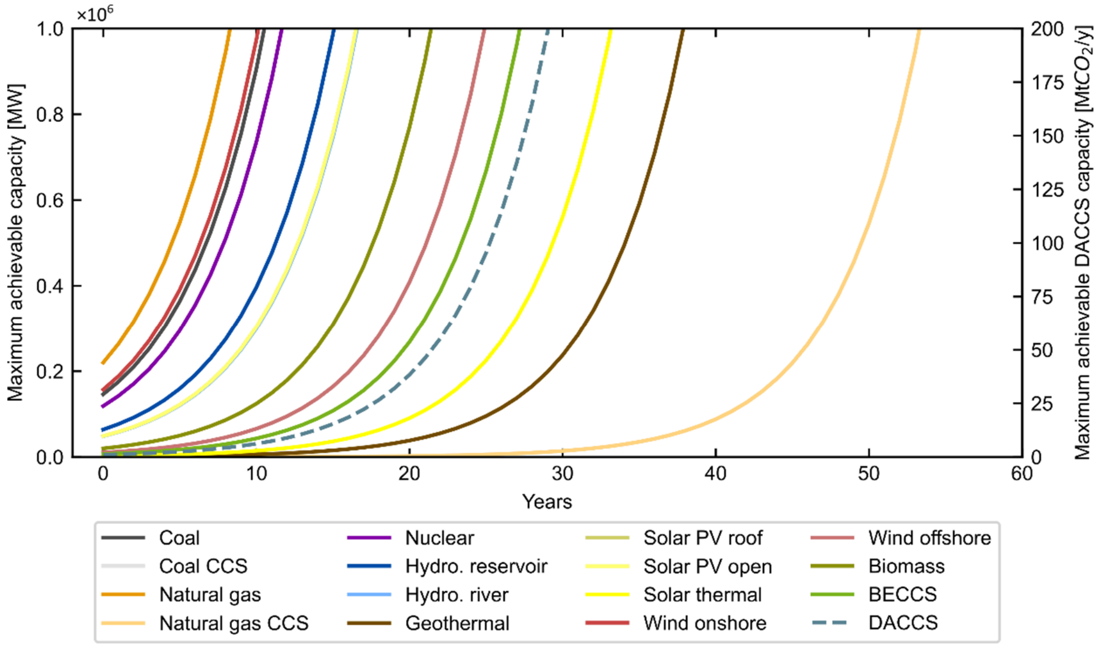

Supplementary Fig. 4. Maximum deployment capacity as a function of time and initial capacity for each technology. *Note the secondary y-axis for direct air carbon capture and storage (DACCS) while bioenergy with carbon capture and storage (BECCS) refers to the primary y-axis.*

The diffusion rate leads to an exponential bound on the capacity, with a small slope in the first years of deployment. For example, a technology with an initial capacity of 60 MW would need 50 years to reach 1 TW, while wind offshore would require 10 years because of its larger initial capacity. Similarly, for DACCS (dashed blue line), it takes around 25 years to scale from a capacity of 1 MtCO_2/yr to a capacity of 200 MtCO_2/yr.

3.5. Regional implications for the SLOW and LATE scenarios

Supplementary Fig. 5 shows the regional power system and the CO_2 emissions removal breakdown for the SLOW scenario, introducing CDR technologies in 2055. **Supplementary Fig. 6** shows the regional power system and the CO_2 emissions removal breakdown for the LATE scenario, introducing CDR technologies in 2055. **Supplementary Fig. 7** shows the trade of biomass, CO_2 and electricity for the SLOW scenario, deploying CDR technologies in 2055. **Supplementary Fig. 8** shows the trade of biomass, CO_2 and electricity for the LATE scenario, introducing CDR technologies in 2085.

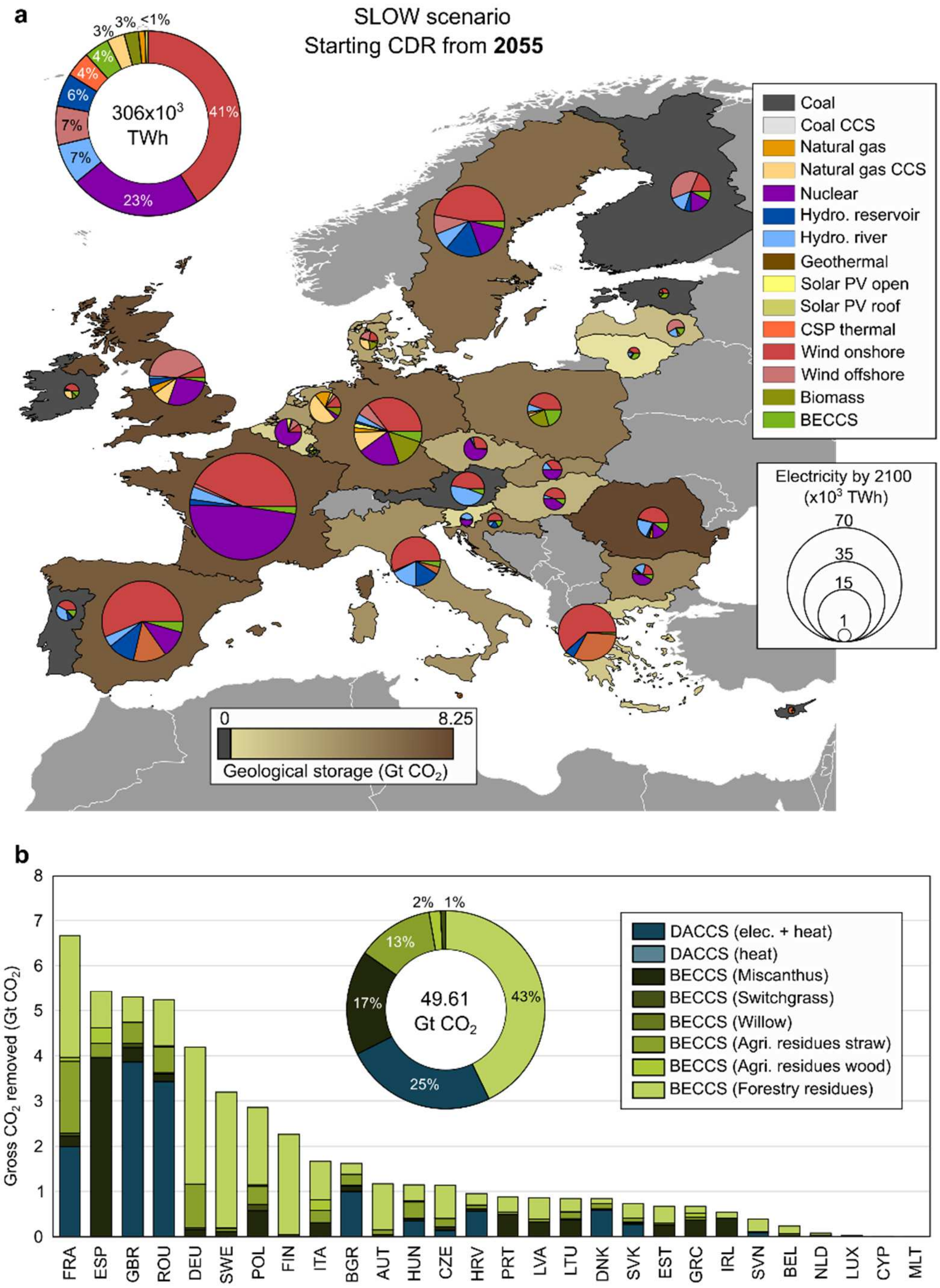

Supplementary Fig. 5**. **Regional implications for the European energy system starting the deployment of bioenergy with carbon capture and storage (BECCS) and direct air carbon capture and storage (DACCS)in 2055** **SLOW** **scenario). *Subplot **a** corresponds to the optimal electricity generation by 2100 in each European country. The pie charts show the share of generation per electricity technology depicted with different colors, while the size of the pie charts is proportional to the generation by 2100 (TWh). Each country is colored according to the CO_2 stored in geological sites; the darker the shade, the greater the CO_2 stored. Subplot **b** shows the breakdown by country of the gross CO_2 removed from the atmosphere considering the different biomass resources for BECCS and DACCS technologies. Countries in subplot **b** are labeled according to the ISO3 code abbreviation. The map in subplot a was created using ArcGIS® 10.7.1 software by Esri[56]; no copyrighted material was used.*

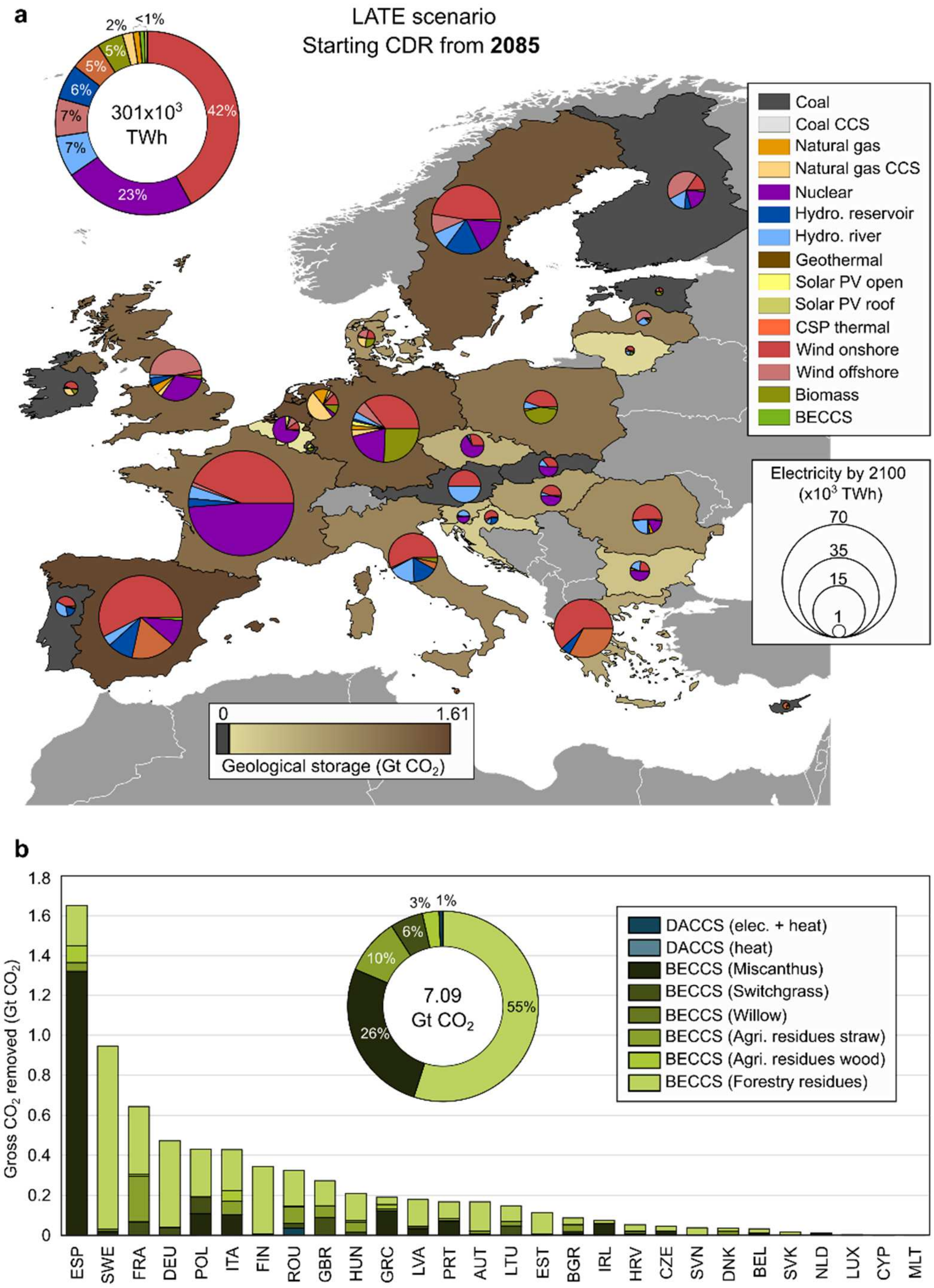

Supplementary Fig. 6. Regional implications for the European energy system starting the deployment of bioenergy with carbon capture and storage (BECCS) and direct air carbon capture and storage (DACCS)in 2085 LATE scenario). *Subplot **a** corresponds to the optimal electricity generation by 2100 in each European country. The pie charts show the share of generation per electricity technology depicted with different colors, while the size of the pie charts is proportional to the generation by 2100 (TWh). Each country is colored according to the CO_2 stored in geological sites; the darker the shade, the greater the CO_2 stored. Subplot **b** shows the breakdown by country of the gross CO_2 removed from the atmosphere considering the different biomass resources for BECCS and DACCS technologies. Countries in subplot **b** are labeled according to the ISO3 code abbreviation. The map in subplot a was created using ArcGIS® 10.7.1 software by Esri[56]; no copyrighted material was used.*

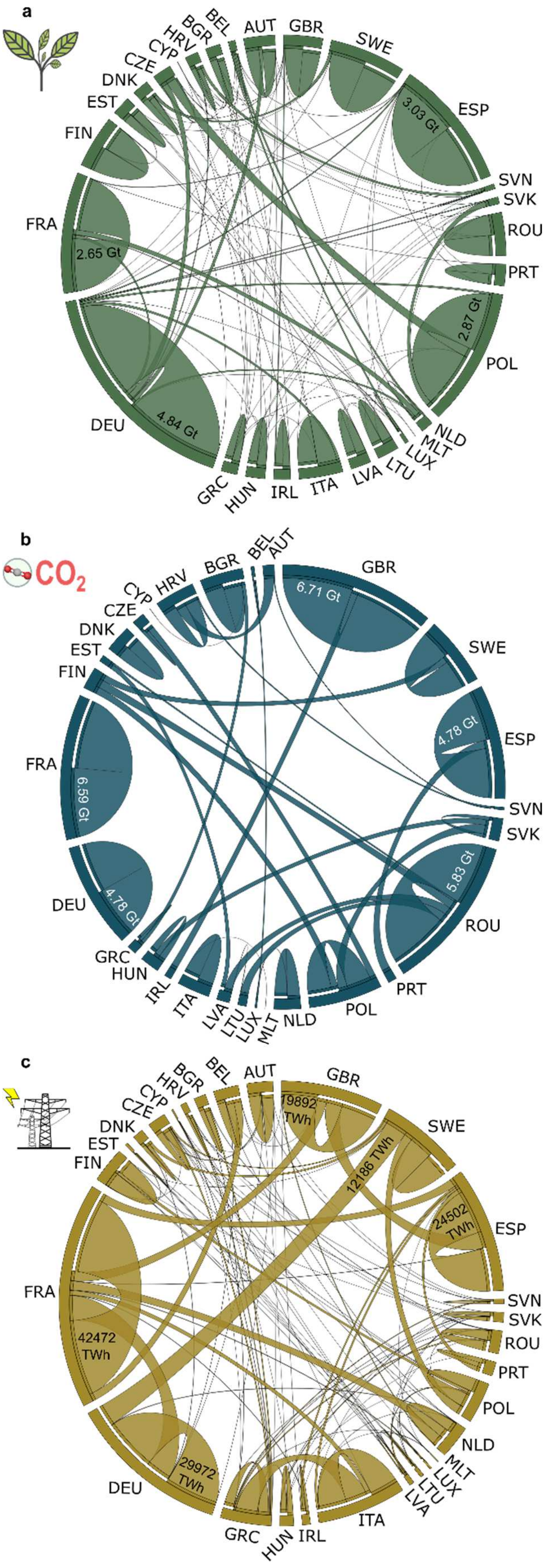

Supplementary Fig. 7. Biomass trade, CO_2 flows and electricity transmission in the SLOW scenario by 2100. *Subplot **a** shows the biomass traded in the form of pellets between European*

*countries. Subplot **b** shows the CO_2 transported via pipeline between European countries. Subplot **c** shows the electricity traded between European countries. In the chord diagrams produced using Circos*[57]*, the European countries are depicted by arcs on the outer part of the circular layout, where the arc length provides the total biomass (subplot **a**), CO_2 (subplot **b**) and electricity (subplot **c**) imported, exported and consumed/stored domestically (the latter refers to chords leaving and entering the same country). Each chord represents a flow, where its thickness is proportional to the magnitude of the trade (some values are indicated for illustrative purposes). Chords directly connected to the countries' arcs represent an export (i.e., exporter country) while those non-connected (separated by a white layer) correspond to imports. Countries are labeled according to the ISO3 code abbreviation.*

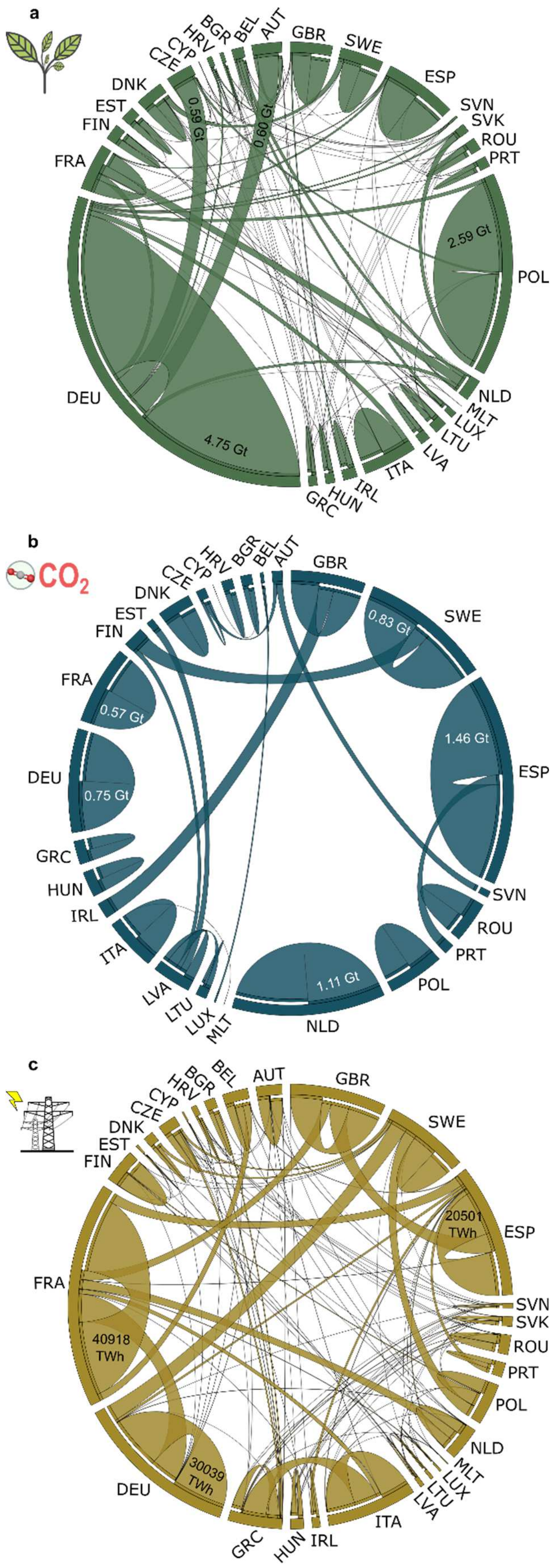

Supplementary Fig. 8. Biomass trade, CO_2 flows and electricity transmission in the LATE scenario by 2100. *Subplot **a** shows the biomass traded in the form of pellets between European*

*countries. Subplot **b** shows the CO_2 transported via pipeline between European countries. Subplot **c** shows the electricity traded between European countries. In the chord diagrams produced using Circos[57], the European countries are depicted by arcs on the outer part of the circular layout, where the arc length provides the total biomass (subplot **a**), CO_2 (subplot **b**) and electricity (subplot **c**) imported, exported and consumed/stored domestically (the latter refers to chords leaving and entering the same country). Each chord represents a flow, where its thickness is proportional to the magnitude of the trade (some values are indicated for illustrative purposes). Chords directly connected to the countries' arcs represent an export (i.e., exporter country) while those non-connected (separated by a white layer) correspond to imports. Countries are labeled according to the ISO3 code abbreviation.*

4. Methodological assumptions and future work

We next highlight the main methodological assumptions in the RAPID modeling framework:

- The RAPID model assumes perfect foresight over the entire horizon, a standard assumption widespread in energy systems models such as TIMES, MARKAL, and MESSAGE[58,59]. In essence, under the perfect foresight assumption, the parameter values during the entire time horizon are assumed to be perfectly known in advance, and the model is solved with full visibility of current and future events. Hence, following the perfect foresight approach, decisions in RAPID are optimized for the entire 2020-2100 horizon, yielding the best possible roadmap based on an ideal planning. The starting year to deploy CDR is defined beforehand, so short-term decisions affecting the power system are optimized with full awareness of longer-term technological and market changes.
  The perfect foresight assumption is fully aligned with our work's goal, which studies the implications of delaying CDR actions by optimizing roadmaps starting from a specific year during the horizon. This perfect foresight approach provides, therefore, a lower bound on the cost and emissions. However, in practice, decision-makers may take short-term decisions with limited information[58,60].
- RAPID adopts a country-level spatial representation. A simplified representation of the EU power system was adopted where the centroids of the countries correspond to demand load areas. Additionally, the capacities installed and resources available refer to these centroids (e.g., biomass residues, marginal land, and geological sites). The biomass and CO_2 storage trades are modeled with arcs between pairs of nodes (centroids) in the resulting network. We assume that all the biomass is converted into pellets and transported via truck. Similarly, CO_2 is always transported via pipeline, as only onshore geological sites are considered. Storage of electricity and biomass between periods is omitted. The costs of the new transmission lines are neglected, yet transportation losses are accounted for. Regarding the temporal representation, RAPID considers a five-year temporal resolution.
  We consider that the temporal and spatial scales are consistent with the goal of this work. A model with higher granularity would most likely lead to the same conclusions and insights, yet it would result in a heavier computational burden.
- The RAPID modeling framework has been initially developed for the EU (27 member countries) plus the United Kingdom, which plays a key role in the European Network of Transmission System Operators for Electricity (ENTSO-E). We focus on assessing the implications of delayed actions on CDR in the EU power system as a highly relevant illustrative case where countries are committed to cooperating to meet the Paris climate goal. We assume full cooperation among countries in terms of electricity transmission,

biomass transportation, and CO_2 trade. We consider the domestic availability of biomass resources (forestry and agricultural residues and marginal land), and onshore geological sites within the EU borders. However, potentials could be increased by considering other residues available (e.g., municipal solid waste) or by adding abandoned agricultural land or land that would be eventually available due to efficiency gains or dietary changes[41]. Similarly, other CO_2 storage alternatives such as offshore geological storage or mineral carbonation could be included. Hence, further research is needed on the regional CO_2 storage capacities to ultimately define the suitability of each specific storage site based on a full range of technical, economic, and environmental constraints.

- Uncertainties in the model arise mainly due to the long-term horizon considered (from 2020 to 2100, consistent with the Paris temperature target). Notably, various parameters in RAPID are inherently uncertain, such as future technology performance, crop yields, and some economic and environmental parameters, among others. To get insight into how these uncertainties affect the results, we performed an *a posteriori* sensitivity analysis of the economic and emissions parameters, providing confidence intervals for the optimal solutions. The uncertainty analysis results for the emissions are shown in Fig. 1b, while the cost results are provided in Supplementary Tables 43-50.
- In RAPID, the emissions balance focuses only on CO_2 emissions. However, other greenhouse gas (GHG) emissions could be incorporated into the model, making the CDR targets more ambitious. Despite globally the methane and nitrous oxide emissions are important contributors to global warming, those GHGs are mostly linked to the livestock and fertilizers in the agricultural sector.
  The life cycle CO_2 emissions for both the foreground and background systems are retrieved from the Ecoinvent v3.5 database[5] accessed through the Simapro software[24]. These emissions data could be adjusted based on prospects on how technologies will evolve in the future under a prospective LCA framework. This approach would lead to more accurate results, yet it would also result in more pronounced uncertainties. As a matter of fact, prospective LCAs are still scarce and could be regarded (to some extent) as proof-of-concept studies, more so when coupled with optimization[61]. As an alternative approach, we carried out a sensitivity analysis to study the effects of uncertainties in the LCA emissions data, which partly stem from technological changes (details in Methods, Uncertainty analysis).

Future research directions of the current work could include:

- The scope of RAPID could be enlarged to consider a broader portfolio of CDR options, including negative emissions technologies and practices (e.g., biochar, soil carbon sequestration, or afforestation/reforestation). Moreover, issues related to the permanence of storage and saturation of sinks, the vulnerability of the CO_2 storage, and the length of crediting horizon should be considered within the scope of the model.
- RAPID could also consider other countries beyond the EU borders and model other high-emitting sectors, e.g., transport, steel industry, heating and building sector, and agriculture. Moreover, other GHG emissions beyond the energy sector could be incorporated in the model, with a focus on hard-to-abate emissions that CDR could offset (e.g., methane emissions from agriculture).
- Other environmental impacts beyond climate change could be incorporated in RAPID, such as impacts on human health or biodiversity. Modeling social or political barriers could also help to reproduce more realistic decision-making environments.

- Uncertainties could be incorporated in RAPID following a stochastic programming or robust optimization framework[62]. This, however, would lead to more complex formulations and larger CPU times.

5. Supplementary references

1. Brooke, A., Kendrick, D., Meeraus, A. & Raman, R. GAMS—A User's Manual. (GAMS Development Corporation, 1998).

2. Iyer, G. et al. Diffusion of low-carbon technologies and the feasibility of long-term climate targets. *Technol. Forecast. Soc. Change* **90**, 103–118 (2015).

3. Dataset Publishing Language, Countries. https://developers.google.com/public-data/docs/canonical/countries_csv

4. Keith, D. W., Holmes, G., Angelo, D. S. & Heidel, K. A Process for Capturing CO_2 from the Atmosphere. *Joule* **2**, 1573–1594 (2018).

5. Wernet, G. et al. The ecoinvent database version 3 (part I): overview and methodology. *Int. J. Life Cycle Assess*. **21**, 1218–1230 (2016).

6. Child, M., Kemfert, C., Bogdanov, D. & Breyer, C. Flexible electricity generation, grid exchange and storage for the transition to a 100% renewable energy system in Europe. *Renew. Energy* **139**, 80–101 (2019).

7. Carlsson, J. E. al. (Ed). ETRI 2014 - Energy Technology Reference Indicator projections for 2010-2050. EUR 26950. Luxembourg (Luxembourg): Publications Office of the European Union; 2014. JRC92496

8. Mathieu, P. *The IPCC special report on carbon dioxide capture and storage*. ECOS 2006 - Proceedings of the 19th International Conference on Efficiency, Cost, Optimization, Simulation and Environmental Impact of Energy Systems (2006).

9. Miranville, A. Annual report 2019. *AIMS Math*. **5**, i–v (2020).

10. Eurostat. Data explorer, 2019. Gas prices for non-household consumers - bi-annual data (from 2007 onwards) (2019).

11. European Commission. Euroatom Supply Agency. Nuclear Observatory Prices. https://ec.europa.eu/euratom/observatory_price.html

12. de Wit, M. & Faaij, A. European biomass resource potential and costs. *Biomass and Bioenergy* **34**, 188–202 (2010).

13. IEA. Greenhouse Gas R&D Programme (IEA GHG), Biomass CCS Study, 2009/9, November 2009

14. Steffen, B. Estimating the cost of capital for renewable energy projects. *Energy Econ*. **88**, 104783 (2020).

15. Zero Emissions Platform. The Costs of CO_2 Transport: Post-demonstration CCS in the EU. European Technology Platform for Zero Emission Fossil Fuel Power Plants, Brussels, Belgium (2011).

16. Fajardy, M. & Mac Dowell, N. Can BECCS deliver sustainable and resource efficient negative emissions? *Energy Environ. Sci.* **10**, 1389–1426 (2017).

17. European Commission. Energy. Data and analysis. Weekly oil bulletin. https://ec.europa.eu/energy/data-analysis/weekly-oil-bulletin_en

18. Budinis, S., Krevor, S., Dowell, N. Mac, Brandon, N. & Hawkes, A. An assessment of CCS costs, barriers and potential. *Energy Strateg. Rev*. **22**, 61–81 (2018).

19. IMF. International Monetary Fund. World Economic Outlook Database April 2020. https://www.imf.org/en/Publications/WEO/weo-database/2020/April

20. Wildbolz, C. Life Cycle Assessment of Selected Technologies for CO_2 Transport and Sequestration. (2007).

21. Camargo, G. G. T., Ryan, M. R. & Richard, T. L. Energy Use and Greenhouse Gas Emissions from Crop Production Using the Farm Energy Analysis Tool. *Bioscience* **63**, 263–273 (2013).

22. ECN.TNO. Phyllis2 - Database for (treated) biomass, algae, feedstocks for biogas production and biochar. https://phyllis.nl/

23. Huijbregts, M. A. J. et al. Framework for modelling data uncertainty in life cycle inventories. *Int. J. Life Cycle Assess*. **6**, 127 (2001).

24. Goedkoop, M., Oele, M., Leijting, J., Ponsioen, T. & Meijer, E. Introduction to LCA with SimaPro. PRé. (2016).

25. Wickwire, S. (2007). Biomass combined heat and power catalog of technologies. US Environmental Protection Agency Combined Heat and Power Partnership, September 2007. https://www. epa. gov/sites/production/files/2015 07/documents/biomass_combined_heat_and_power_catalog_of_technologies_v, 1, 122.

26. Fajardy, M., Chiquier, S. & Mac Dowell, N. Investigating the BECCS resource nexus: delivering sustainable negative emissions. *Energy Environ. Sci*. **11**, 3408-3430 (2018).

27. Vangkilde-Pedersen, T. et al. Assessing European capacity for geological storage of carbon dioxide–the EU GeoCapacity project. *Energy Procedia* **1**, 2663–2670 (2009).

28. Mortensen, G. M., Bergmo, P. E. S. & Emmel, B. U. Characterization and estimation of CO_2 storage capacity for the most prospective aquifers in Sweden. *Energy Procedia* **86**, 352–360 (2016).

29. European Commission. Directorate-General for Energy. (2019). EU Energy in Figures: Statistical Pocketbook. Publications Office of the European Union.

30 . Capros, P. et al 2016 EU Reference Scenario 2016. Energy Transport and GHG Emissions–Trends to 2050 (Luxembourg: Publication Office of the European Union) (2016)

31. Ruiz, P. et al. ENSPRESO-an open, EU-28 wide, transparent and coherent database of wind, solar and biomass energy potentials. *Energy Strateg. Rev*. **26**, 100379 (2019).

32. Adam, K., Müller-Mienack, M., Paun, M., Sanchis, G. & Strunz, K. e-HIGHWAY 2050—The ENTSO-E facilitated study programme towards a Modular Development Plan on pan-European Electricity Highways System 2050. in IEEE Power and Energy Society General Meeting (2012).

33. Zurano-Cervelló, P., Pozo, C., Mateo-Sanz, J. M., Jiménez, L. & Guillén-Gosálbez, G. Sustainability efficiency assessment of the electricity mix of the 28 EU member countries combining data envelopment analysis and optimized projections. *Energy Policy* **134**, 110921 (2019).

34. Van Wees, J. D., et al. "A prospective study on the geothermal potential in the EU." *Geoelect Report* 13 (2013).

35. Pirker, O. et al. Hydro in Europe: Powering renewables. Renew. Action Plan 66 (2011).

36. EREC: European Renewable Energy Council. Mapping Renewable Energy Pathways towards 2020. Eur. Renew. Energy Counc. 28 (2011).

37. Swart, R. J., et al. Europe's onshore and offshore wind energy potential: An assessment of environmental and economic constraints. No. 6/2009. European Environment Agency, 2009.

38. Pozo, C., Galán-Martín, Á., Reiner, D. M., Mac Dowell, N. & Guillén-Gosálbez, G. Equity in allocating carbon dioxide removal quotas. *Nat. Clim. Chang.* **10**, 640–646 (2020).

39. Cai, X., Zhang, X. & Wang, D. Land availability for biofuel production. *Environ. Sci. Technol*. **45**, 334–339 (2010).

40. Fritz, S. et al. Downgrading recent estimates of land available for biofuel production. *Environ. Sci. Technol*. **47**, 1688–1694 (2013).

41. Röös, E. et al. Greedy or needy? Land use and climate impacts of food in 2050 under different livestock futures. *Glob. Environ. Chang*. **47**, 1–12 (2017).

42. Plessmann, G. & Blechinger, P. How to meet EU GHG emission reduction targets? A model based decarbonization pathway for Europe's electricity supply system until 2050. *Energy Strateg. Rev*. **15**, 19–32 (2017).

43. Pietzcker, R. C., Osorio, S. & Rodrigues, R. Tightening EU ETS targets in line with the European Green Deal: Impacts on the decarbonization of the EU power sector. *Appl. Energy* **293**, 116914 (2021).

44. Wietschel, L., Thorenz, A. & Tuma, A. Spatially explicit forecast of feedstock potentials for second generation bioconversion industry from the EU agricultural sector until the year 2030. *J. Clean. Prod*. **209**, 1533–1544 (2019).

45. Dyjakon, A. & García-Galindo, D. Implementing Agricultural Pruning to Energy in Europe: Technical, Economic and Implementation Potentials. *Energies* **12**, 1513 (2019).

46. IINAS - International Institute for Sustainability Analysis and Strategy EFI - European Forest Institute, and J.-J. R. Forest biomass for energy in the EU: current trends, carbon balance and sustainable potential for BirdLife Europe, EEB, and Transport & Environment. 1–121 (2014).

47. ENTSOE. Central collection and publication of electricity generation, transportation and consumption data and information for the pan-European market. Available at: https://transparency.entsoe.eu/generation/r2/installedGenerationCapacityAggregation/show.

48. EUROSTAT. Electricity production capacities by main fuel groups and operator. Available at: https://ec.europa.eu/eurostat/cache/metadata/en/nrg_inf_epc_esms.htm.

49. IRENA. Renewable capacity statistics 2020 International Renewable Energy Agency (IRENA). (2020).

50. Enemalta. Enemalta coorporation. Delimara Power Station. Available at: https://www.enemalta.com.mt/about-us/delimara-power-station/.

51. EurObserver. Solar thermal and concentrated solar power barometer. (2019).

52. Murdock, Hannah E., et al. Renewables 2020-Global status report. (2020). https://www.iea.org/reports/renewables-2020.

53. Galán-Martín, A. et al. Time for global action: an optimised cooperative approach towards effective climate change mitigation. *Energy Environ. Sci.* **11**, 572-581 (2018).

54. Trieb, F. et al. Captive and Open Sea Energy Import Framework. REACCESS Deliverable D2.1 & D3.1. (2010).

55. IEA. Natural Gas Information: Database Documentation. (2019).

56. ESRI (2019). ArcGIS Desktop: Release 10.7.1. Redlands, CA (https://desktop.arcgis.com/en/arcmap)

57. Krzywinski, M. et al. Circos: an information aesthetic for comparative genomics. *Genome Res*. **19**, 1639–1645 (2009).

58. Nerini, F. F., Keppo, I. & Strachan, N. Myopic decision making in energy system decarbonisation pathways. A UK case study. *Energy Strateg. Rev*. **17**, 19–26 (2017).

59. Keppo, I. & Strubegger, M. Short term decisions for long term problems–The effect of foresight on model based energy systems analysis. *Energy* **35**, 2033–2042 (2010).

60. Heuberger, C. F., Staffell, I., Shah, N. & Mac Dowell, N. Impact of myopic decision-making and disruptive events in power systems planning. *Nat. Energy* **3**, 634–640 (2018).

61. Mendoza Beltran, A. et al. When the background matters: using scenarios from integrated assessment models in prospective life cycle assessment. *J. Ind. Ecol*. **24**, 64–79 (2020).

62.Sahinidis, N. V.. Optimization under uncertainty: state-of-the-art and opportunities. *Comput. Chem. Eng.*, **28**(6-7), 971-983 (2004).
