# Supplementary material for: Delaying carbon dioxide removal in the European Union puts climate targets at risk
Source: Nat Commun. 2021 Nov 11;12:6490. doi: 10.1038/s41467-021-26680-3 (PMC8586243; doi:10.1038/s41467-021-26680-3)
Supplement: Supplementary file 4 — Description of Additional Supplementary Files [file 41467_2021_26680_MOESM4_ESM.docx]

Description of Additional Supplementary Files

Title: Supplementary Data 1

Description: This file contains all data supporting the findings of this study.
